# TIE2 activation by antibody-clustered endogenous angiopoietin-2 prevents capillary loss and fibrosis in experimental kidney disease

**DOI:** 10.1172/JCI190286

**Published:** 2025-09-15

**Authors:** Riikka Pietilä, Amanda Marks-Hultström, Liqun He, Sami Nanavazadeh, Susan E. Quaggin, Christer Betsholtz, Marie Jeansson

**Affiliations:** 1 Department of Immunology, Genetics and Pathology, Uppsala University, Uppsala, Sweden.; 2 Feinberg Cardiovascular Research Institute and Division of Nephrology and Hypertension, Northwestern University, Chicago, Illinois, USA.; 3 Department of Medicine Huddinge, Karolinska Institutet, Huddinge, Sweden.

**Keywords:** Nephrology, Vascular biology, Chronic kidney disease, Drug therapy, Fibrosis

## Abstract

The role of endothelial dysfunction in tubulointerstitial fibrosis associated with chronic kidney disease (CKD) is not well understood. In this study, we demonstrate that the activation of the endothelial tyrosine kinase TIE2 alleviates renal pathology in experimental CKD in mice. TIE2 activation was achieved using a human angiopoietin-2–binding and TIE2-activating antibody (ABTAA) or through adult-induced endothelium-specific knockout of the vascular endothelial protein tyrosine phosphatase gene (*Veptp*). Both methods markedly protected CKD mice from endothelial dysfunction, peritubular capillary loss, tubular epithelial injury, and tubulointerstitial fibrosis. Conversely, silencing TIE2 through adult-induced endothelium-specific knockout of the *Tie2* gene exacerbated CKD pathology. Additionally, we found that endothelial dysfunction promoted renal fibrosis not through endothelial-to-mesenchymal transition, as previously expected, but by inducing the expression of profibrotic PDGFB in tubular epithelial cells, a process that is inhibited by TIE2 activation. Our findings suggest that TIE2 activation via ABTAA warrants investigation as a therapy in human CKD, where there is a substantial unmet medical need.

## Abstract 2

Antibody-mediated TIE2 activation by endogeneous angiopoietin-2 prevents vascular injury and ensuing tubular injury and tubulointerstitial fibrosis in experimental chronic kidney disease.

## Graphical abstract

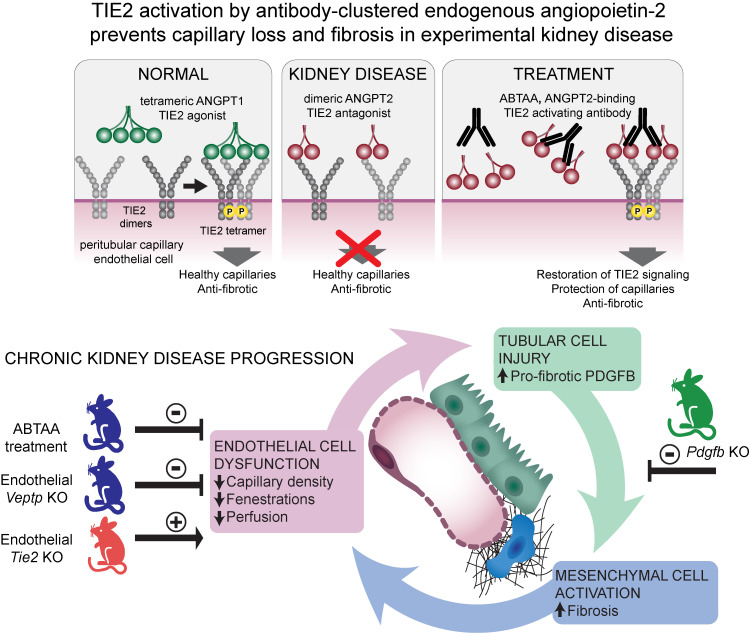

## Introduction

Chronic kidney disease (CKD) affects approximately 13% of the global population and is a leading cause of death due to cardiovascular disease (1–3). It is projected that CKD will rank as the fifth leading cause of death by 2040 (4). While various conditions, such as diabetes and hypertension, can lead to CKD, the common pathogenic pathway typically involves endothelial dysfunction, capillary rarefaction, tubular atrophy, and tubulointerstitial fibrosis, as observed in both animal models and patients (5–18). With no cure available and only a limited number of therapies to slow its progression, there is an urgent need to identify and develop new and effective treatments against CKD. Despite extensive research into the mechanism underlying renal fibrosis, the role and dysregulation of the vasculature, as well as its contributions to tubular injury and fibrosis, remain inadequately understood.

TIE2, also known as TEK, is a tyrosine kinase receptor primarily expressed by endothelial cells. The phosphorylation of TIE2 and thereby triggered downstream signaling pathways promote vascular quiescence and reduce vascular permeability, inflammation, and endothelial apoptosis (19–21). The activity of TIE2 is regulated by angiopoietin (ANGPT) ligands, specifically ANGPT1 and ANGPT2, as well as the vascular endothelial protein tyrosine phosphatase VEPTP. Research has shown that ANGPT1, the primary agonist for TIE2, is decreased in both CKD patients and experimental models (5, 22–26). Conditional deletion of *Angpt1* has resulted in more severe kidney injury in cases of diabetes and obstruction-induced CKD (5, 24). Conversely, circulating levels of ANGPT2, which acts as a context-dependent antagonist of TIE2, increase in CKD patients and correlate with adverse cardiovascular and renal outcomes (27–31). Inhibition of ANGPT2 has recently been shown to reduce kidney injury in experimental CKD (27).

VEPTP is a phosphatase that decreases tyrosine phosphorylation of several proteins, including TIE2 (32, 33). VEPTP is upregulated in experimental models of diabetes and ischemia/reperfusion injury, and conditional knockout of *Veptp* has been found to increase the activity of TIE2 and reduce kidney injury (34, 35). ANGPT1, as well as ANGPT1 mimetics and transgenic expression of *Angpt1*, have demonstrated beneficial effects in various preclinical models of kidney injury (23, 25, 26, 35–38). However, limitations related to production, storage, half-life, applicability, and efficacy of these compounds have hindered their clinical use in CKD. ABTAA is an ANGPT2-binding and TIE2-activating antibody (39) that clusters endogenous ANGPT2 into a complex, enabling TIE2 activation and vascular normalization. ABTAA has shown protective effects in sepsis-induced kidney injury (39), causes tumor vessel normalization (40), and promotes choriocapillary regeneration in macular degeneration (41). Notably, ABTAA offered greater protection in sepsis compared with ANGPT2 inhibition alone, underscoring its dual functional advantage (39).

While it is widely recognized that both endothelial dysfunction and tubulointerstitial fibrosis occur in CKD, the relationship between the two remains unclear. Tubulointerstitial fibrosis develops when perivascular mesenchymal cells, normally responsible for structurally and functionally supporting capillaries (42), become activated, proliferate, migrate, and deposit extracellular matrix (ECM) (43–47). PDGF-B (PDGFB) acts as a mitogen for perivascular mesenchymal cells expressing the PDGF receptor-β (PDGFRB) and is necessary for the proper recruitment and maintenance of pericytes around capillaries (42, 48). PDGFB/PDGFRB signaling has been associated with fibrosis; for example, constitutive activation of PDGFRB in mesenchymal cells has been shown to result in spontaneous renal fibrosis (49), while inhibition of PDGFB or PDGFRB has reduced fibrosis in experimental CKD models (50–56). A highly debated mechanism for the development of renal fibrosis is the notion that activated mesenchymal cells originate from endothelial-to-mesenchymal transition (EndoMT) (57).

In this study, we investigated how endothelial dysfunction and inactivation of TIE2 signaling in CKD affect tubular injury and PDGFB-driven tubulointerstitial fibrosis. Additionally, we explored whether treatment with ABTAA could serve as an effective therapy for renal injury associated with experimental CKD.

## Results

### Pharmacological or genetic activation of TIE2 improves renal perfusion and prevents capillary rarefaction following experimental CKD.

**Figure 1:**
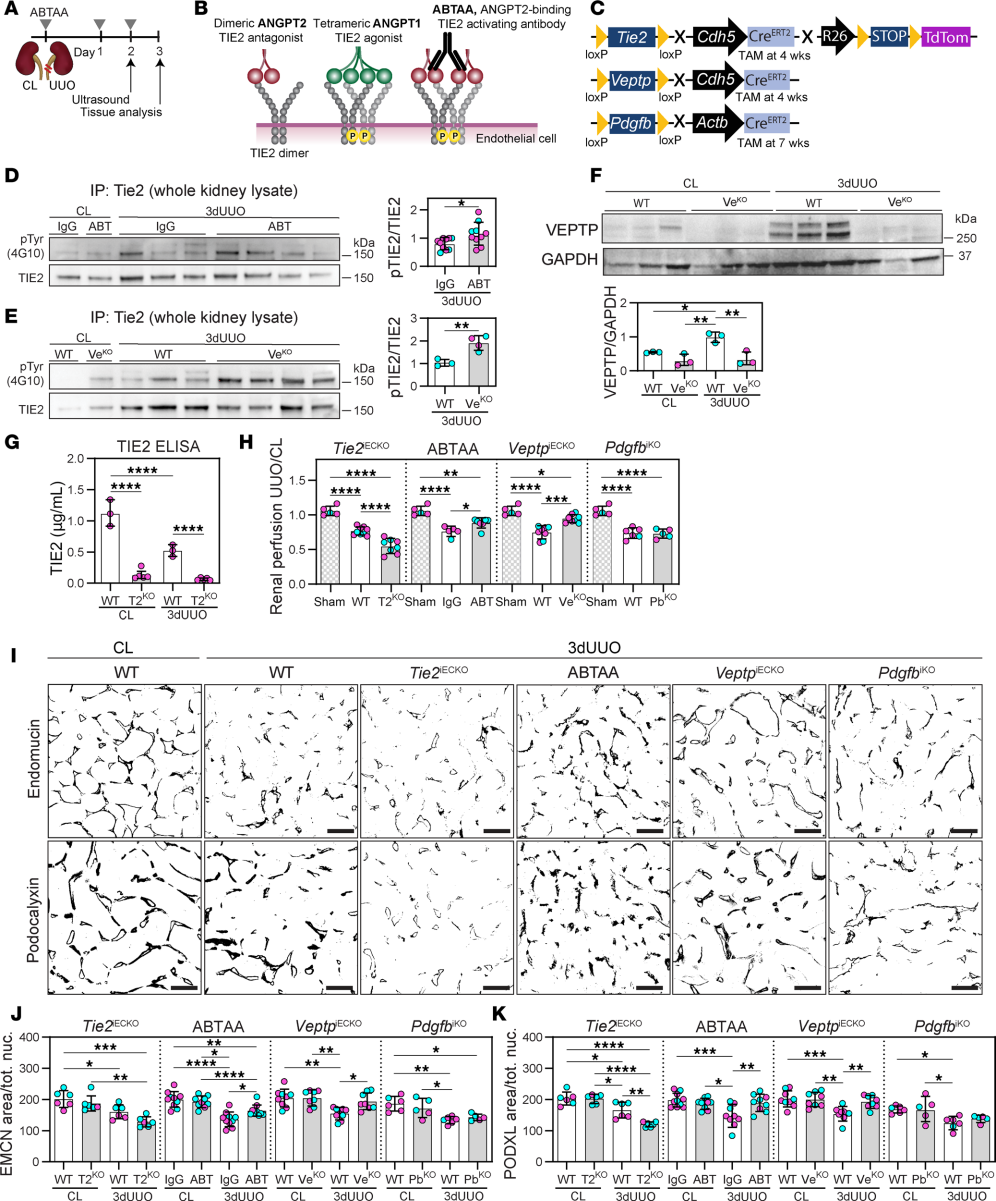
Pharmacological or genetic activation of TIE2 improves renal perfusion and prevents capillary rarefaction following experimental CKD. (**A**) Schematic diagram of the experimental setup with UUO, contralateral control kidney (CL), and ABTAA (or IgG) administration. (**B** and **C**) Schematic diagram of ABTAA function (**B**) and inducible conditional mouse lines used in the study (**C**). TAM, tamoxifen induction. (**D** and **E**) pTIE2/TIE2 in 3-day UUO kidneys in ABTAA (ABT) and *Veptp*^iECKO^ (Ve^KO^) mice, respectively. Data are based on *n* = 3–10 mice/group and 3 (**D**) and 1 (**E**) blots (see Supplemental Figure 2). (**F**) VEPTP in kidney lysates from CL and 3-day UUO kidneys in WT and *Veptp*^iECKO^ mice. Data are based on *n* = 3 mice/group and 1 blot (see Supplemental Figure 2). (**G**) TIE2 protein measured by ELISA in kidney homogenates from CL and 3-day UUO kidneys in *Tie2*^iECKO^ (T2^KO^) mice. Data are based on *n* = 3–5 mice/group. (**H**) Renal perfusion measured with ultrasound contrast imaging in 2-day UUO/CL kidneys from ABTAA-treated, *Veptp*^iECKO^, *Tie2*^iECKO^, and *Pdgfb*^iKO^ (Pb^KO^) mice and their respective controls. The same group of sham-operated mice was used to establish baseline perfusion levels for comparison with UUO-injured kidneys. Data are based on *n* = 5–8 mice/group. (**I**–**K**) Immunohistochemistry and quantification from renal cortex for endothelial markers (endomucin [EMCN] and podocalyxin [PODXL]) in 3-day UUO kidneys from ABTAA-treated, *Veptp*^iECKO^, *Tie2*^iECKO^, and *Pdgfb*^iKO^ mice and their controls. Data are based on *n* = 5–11 mice/group and >600 images/marker. Scale bars: 50 μm. Data represent mean ± SD, and each symbol represents 1 mouse (females, magenta; males, cyan). *P < 0.05, **P < 0.01, ***P < 0.001, and ****P < 0.0001, determined by 1-way ANOVA and Tukey’s post hoc test (**F**–**H**, **J**, and **K**) or unpaired 2-tailed Student’s *t* test (**D** and **E**).

We investigated the role of TIE2 signaling through gain- and loss-of-function experiments utilizing the unilateral ureter obstruction (UUO) model, a widely used progressive model of CKD (5). UUO injury is characterized by capillary rarefaction and tubulointerstitial fibrosis (5, 16, 58) in the obstructed kidney, while the contralateral kidney serves as a control (Figure 1A). ABTAA treatment or conditional endothelial knockout of *Veptp* (*Veptp*^iECKO^) was employed to induce TIE2 activation (Figure 1, A–C). In contrast, conditional endothelial knockout of *Tie2* (*Tie2*^iECKO^) was used as a model of TIE2-incompetent signaling. We investigated the causal effect of PDGFB on tubulointerstitial fibrosis using a conditional global knockout of *Pdgfb* (*Pdgfb*^iKO^) (Figure 1C).

ABTAA requires ANGPT2 to activate TIE2 (39), and increased levels of ANGPT2 were confirmed in UUO kidneys (Supplemental Figure 1, A and B; supplemental material available online with this article; https://doi.org/10.1172/JCI190286DS1). However, circulating ANGPT2 levels remained unchanged, indicating that the injury and resulting ANGPT2 expression are localized to UUO kidneys (Supplemental Figure 1C). Both ABTAA-treated and *Veptp*^iECKO^ mice exhibited increased TIE2 phosphorylation in the UUO kidneys (Figure 1, D and E, and Supplemental Figure 2). Importantly, ABTAA did not induce systemic TIE2 activation, as demonstrated by the unchanged TIE2 phosphorylation levels in lung tissue (Supplemental Figure 1D), contrasting with the broad effects observed in *Veptp*^iECKO^ (34). UUO resulted in increased VEPTP levels and decreased TIE2 expression (Figure 1, F and G). Both *Veptp*^iECKO^ and *Tie2*^iECKO^ showed significant reductions in their respective protein expression (Figure 1, F and G, and Supplemental Figure 3).

Renal perfusion was assessed using contrast-enhanced ultrasound imaging. The UUO-induced reduction in renal perfusion was significantly prevented in ABTAA-treated and *Veptp*^iECKO^ mice compared with IgG-treated and WT controls. The *Pdgfb*^iKO^ did not affect renal perfusion. In contrast, the *Tie2*^iECKO^ mice exhibited significantly lower UUO-induced renal perfusion compared with WT mice (Figure 1H). The disadvantage in *Tie2*^iECKO^ was apparent after just 1 day of UUO, where microangiopathy indicated a significant reduction in perfused capillaries in *Tie2*^iECKO^ mice but not in WT mice (Supplemental Figure 4, A and B).

Peritubular capillary (PTC) density in the renal cortex was evaluated using immunostaining for the endothelial markers endomucin and podocalyxin (59). UUO-induced PTC rarefaction was significant in the kidneys subjected to 3-day UUO compared with the contralateral kidneys. Both ABTAA treatment and *Veptp*^iECKO^ significantly prevented the loss of PTCs, while *Tie2*^iECKO^ mice experienced exacerbated capillary rarefaction. The *Pdgfb*^iKO^ did not influence UUO-induced capillary rarefaction (Figure 1, I–K).

### Pharmacological or genetic TIE2 activation prevents UUO-induced endothelial injury.

**Figure 2:**
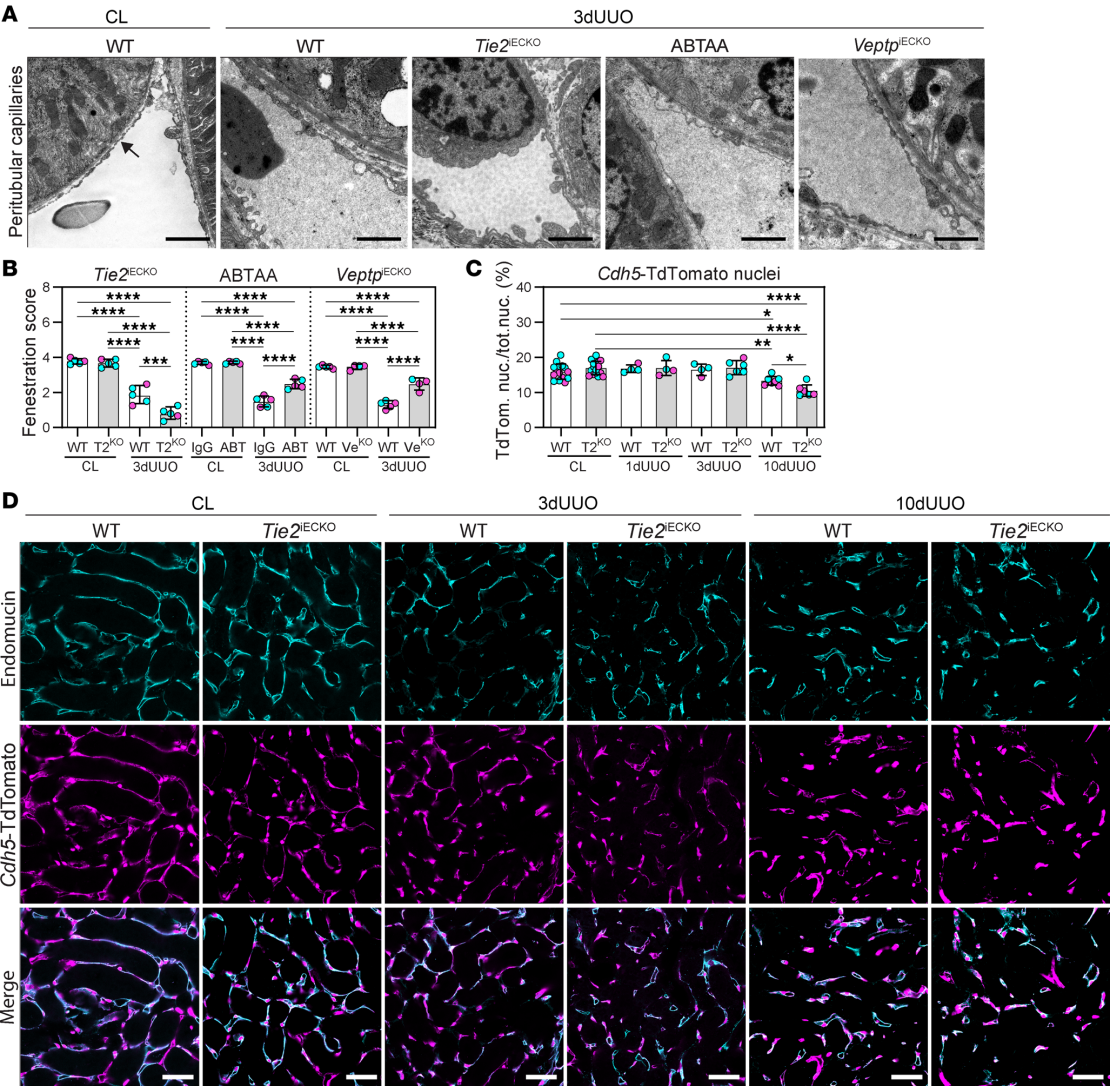
Pharmacological or genetic TIE2 activation prevents UUO-induced endothelial injury. (**A** and **B**) Semiquantitative grading of capillary length with fenestrations from micrographs of peritubular capillaries in 3-day UUO kidneys from ABTAA-treated (ABT), *Veptp*^iECKO^ (Ve^KO^), and *Tie2*^iECKO^ (T2^KO^) mice. Data are based on *n* = 4–5 mice/group and scoring of 892 micrographs. Scoring is based on percentage of endothelium with fenestrations as follows: 0, 0%–5%; 1, 6%–25%; 2, 26%–50%; 3, 51%–75%; and 4, 76%–100%. The fenestrated endothelium is indicated by an arrow. Scale bars: 2 μm. (**C**) Quantification of endothelial nuclei in *Tie2*^iECKO^ and WT mice from 1-, 3-, and 10-day UUO kidneys. Data are based on *n* = 4–7 mice/group, and 310 images were quantified. (**D**) Representative image with immunohistochemistry for endomucin (cyan) together with the endothelial *Cdh5*-Tdtomato lineage tracer (magenta). Scale bars: 50 μm. Data represent mean ± SD, and each symbol represents 1 mouse (females, magenta; males, cyan). *P < 0.05, **P < 0.01, ***P < 0.001, and ****P < 0.0001, determined by 1-way ANOVA and Tukey’s post hoc test (**B** and **C**).

Fenestrations in PTCs are essential structures for reabsorption of water and solutes from the tubular system. These fenestrations are known to be reduced in CKD and can serve as indicators of capillary dysfunction (16). In the contralateral kidneys, all groups of animals displayed healthy, continuous fenestrations of PTCs (Figure 2, A and B). The UUO-induced reduction in fenestrations was significantly prevented in ABTAA-treated and *Veptp*^iECKO^ mice compared with IgG-treated and WT controls. In contrast, the UUO kidneys in *Tie2*^iECKO^ mice exhibited a significantly greater loss of fenestrations compared with UUO kidneys in WT mice (Figure 2, A and B).

UUO injury had no impact on glomerular vessels and podocytes in WT and *Tie2*^iECKO^ mice (Supplemental Figure 5), as previously reported (60). Despite the evident capillary rarefaction observed in kidneys subjected to 3 days of UUO, endothelial cells remained present in the tissue following the injury. Endothelial nuclei labeled with lineage tracer *Cdh5*-TdTomato remained consistent in kidneys after 3 days of UUO (Figure 2C), indicating that the reduced capillary density does not merely reflect endothelial cell death. However, 10-day UUO kidneys showed a significant loss of endothelial nuclei in the cortex, with a further reduction in *Tie2*^iECKO^ mice (Figure 2C). This reduction was not a result of altered cell proliferation, as both Ki67 staining and EdU incorporation resulted in similar responses in WT and *Tie2*^iECKO^ mice (Supplemental Figure 6, A–D). Furthermore, the *Cdh5*-TdTomato lineage reporter overlapped with endomucin staining in PTCs of both WT and *Tie2*^iECKO^ mice up to 10 days after UUO (Figure 2D).

### Pharmacological or genetic TIE2 activation prevents UUO-induced tubulointerstitial fibrosis.

**Figure 3:**
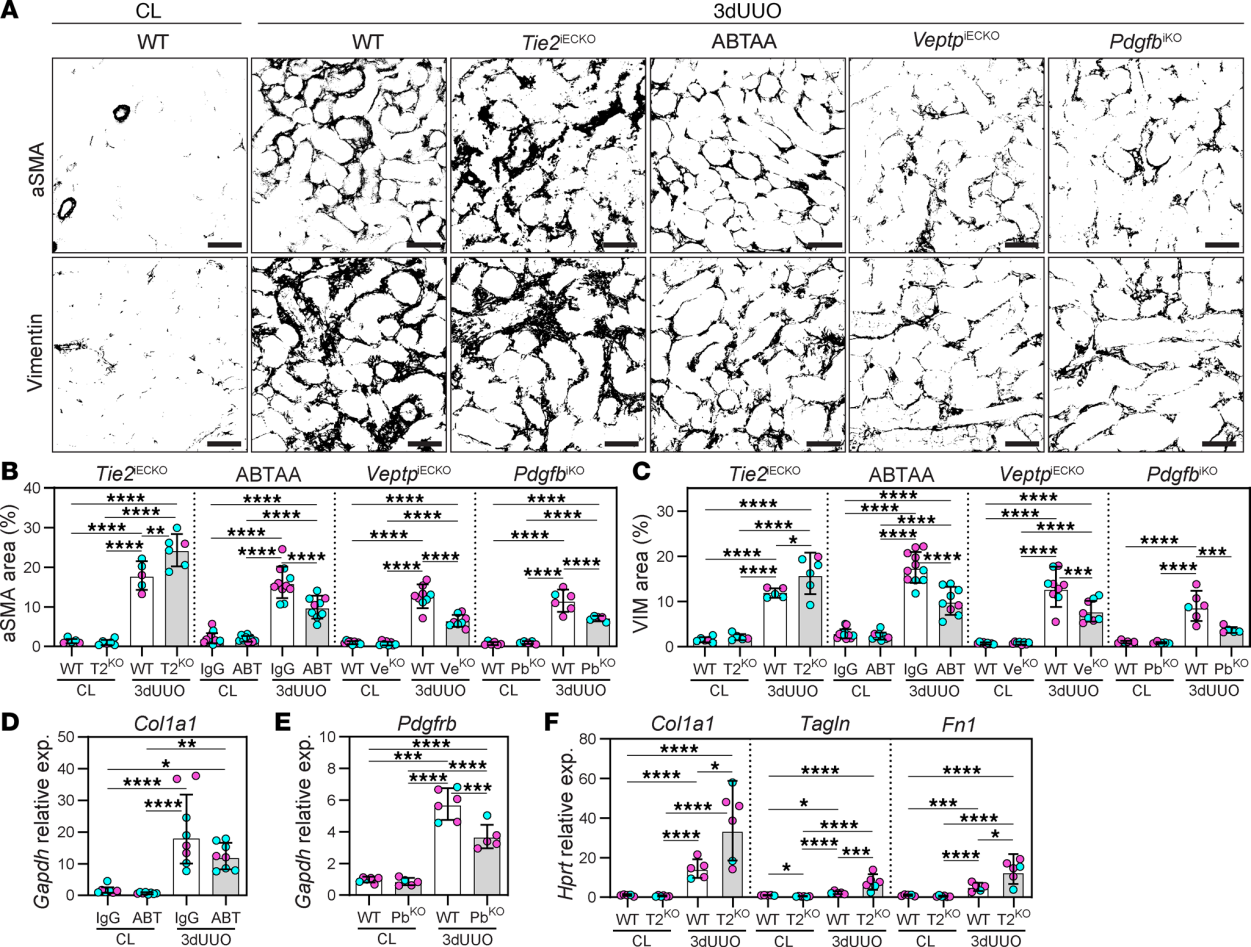
Pharmacological or genetic TIE2 activation prevents UUO-induced tubulointerstitial fibrosis. (**A**–**C**) Immunohistochemistry and quantifications from renal cortex for fibrosis markers (aSMA and vimentin [VIM]) in 3-day UUO kidneys from ABTAA-treated (ABT), *Veptp*^iECKO^ (Ve^KO^), *Tie2*^iECKO^ (T2^KO^), and *Pdgfb*^iKO^ (Pb^KO^) mice and their controls. Data are based on *n* = 5–11 mice/group and quantifications from >600 images/marker. Scale bars: 50 μm. (**D**) Renal *Col1a1* expression in ABTAA-treated mice. Data are based on *n* = 6 mice/group. (**E**) Renal *Pdgfrb* expression in *Pdgfb*^iKO^ mice. Data are based on *n* = 5–6 mice/group. (**F**) Gene expression of *Col1a1*, *Tagln*, and *Fn1* in 3-day UUO kidneys from *Tie2*^iECKO^ mice. Data are based in *n* = 5–6 mice/group. Data represent mean ± SD, and each symbol represents 1 mouse (females, magenta; males, cyan). *P < 0.05, **P < 0.01, ***P < 0.001, and ****P < 0.0001, determined by 1-way ANOVA and Tukey’s post hoc test (**B**–**F**).

The activation of perivascular mesenchymal cells into myofibroblasts is characterized by the expression of α-smooth muscle actin (aSMA) and vimentin, as well as the production of ECM components, including type I collagen and fibronectin (61, 62). Fibrosis in the renal cortex was quantified by staining for aSMA and vimentin. UUO-induced fibrosis was significantly reduced in ABTAA-treated and *Veptp*^iECKO^ mice compared with controls (Figure 3, A–C). A similar trend (reduction) was noted for *Col1a1* and *Pdgfrb* in ABTAA-treated and *Pdgfb*^iKO^ mice, respectively (Figure 3, D and E). Additionally, *Pdgfb*^iKO^ mice exhibited significantly reduced fibrosis (Figure 3, A–C), supporting the role of PDGFB as a major mesenchymal cell activator following UUO. In contrast, *Tie2*^iECKO^ mice displayed markedly more fibrosis in their 3-day UUO kidneys, along with increased expression of *Col1a1*, *Tagln*, and *Fn1* (Figure 3F).

Collectively, our data demonstrate that the protective role of ABTAA on vascular integrity also effectively reduces tubulointerstitial fibrosis, the final pathological process in CKD.

### UUO-induced tubulointerstitial fibrosis is not the result of EndoMT.

**Figure 4:**
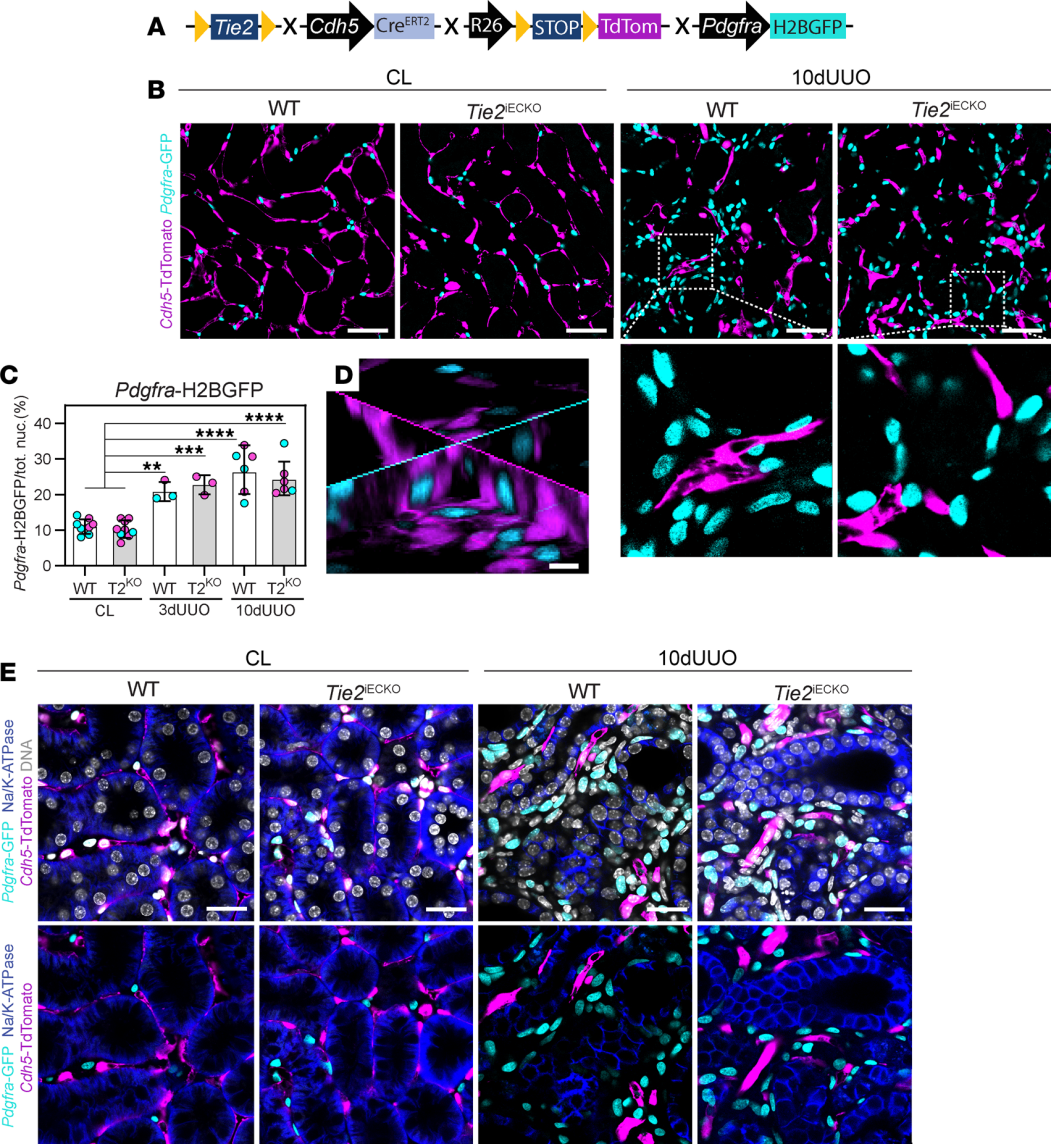
UUO-induced tubulointerstitial fibrosis is not the result of EndoMT. (**A**) Breeding strategy for *Tie2*^iECKO^ mice with endothelial lineage tracer and myofibroblast reporter. (**B** and **C**) Imaging and quantification of *Pdgfra*-H2BGFP^+^ nuclei (cyan) in renal cortex of 3- and 10-day UUO kidneys in WT and *Tie2*^iECKO^ (T2^KO^) mice. Data are based on *n* = 3–6 mice/group and quantifications from >180 images. Scale bars: 50 μm. (**D**) Representative confocal image stack (30 μm) from renal cortex showing *Cdh5*-TdTomato (magenta) and *Pdgfra*-H2BGFP (cyan) in renal cortex 10 days after UUO in a *Tie2*^iECKO^ mouse. Scale bar: 10 μm. (**E**) Confocal images from renal cortex showing tubular epithelial marker NaK/ATPase (blue), *Pdgfra*-GFP (cyan), and *Cdh5*-TdTomato (magenta) 10 days after UUO in WT and Tie2^iECKO^ mice. Representative images of *n* = 3 mice per group. Scale bars: 25 μm. Data in graphs represent mean ± SD, and each symbol represents 1 mouse (females, magenta; males, cyan). **P < 0.01, ***P < 0.001, and ****P < 0.0001, determined by 1-way ANOVA and Tukey’s post hoc test (**C**).

EndoMT has been suggested to contribute to fibrosis in CKD models including UUO (63). To investigate this, we introduced a fibroblast-specific nuclear *Pdgfra*-H2BGFP (64) allele into the *Tie2*^iECKO^ line, resulting in WT and *Tie2*^iECKO^ mice that had both an endothelium-specific linage reporter (*Cdh5*-TdTomato) and a mesenchymal/myofibroblast reporter (nucleus-located GFP) (Figure 4A). *Tie2*^iECKO^ mice were included in the experiment because they exhibit worse endothelial injury after UUO than WT mice, thus representing a model with more unstable endothelial cells. RNA-ISH for *Pdgfra* and *Pdgfrb* revealed that mesenchymal cells responding to UUO are positive for both markers, suggesting that fibrosis is primarily driven by dual-expressing *Pdgfra*/*Pdgfrb* cells (Supplemental Figure 6F).

As expected, the number of *Pdgfra*-H2BGFP^+^ cells increased significantly in the renal cortex of kidneys subjected to 3 and 10 days of UUO. However, no differences were observed between WT and *Tie2*^iECKO^ mice in the number of *Pdgfra*-H2BGFP^+^ cells 3 or 10 days after UUO (Figure 4, B and C). These findings suggest that the increased tubulointerstitial fibrosis seen during disease progression and in *Tie2*^iECKO^ mice reflects a change in the activation state of mesenchymal cells rather than a difference in their quantity. Importantly, no *Cdh5*-TdTomato^+^/*Pdgfra*-H2BGFP^+^ cells were identified in either group, providing no evidence of UUO-induced EndoMT in WT or *Tie2*^iECKO^ mice (Figure 4, B and D). Additionally, tubular epithelial cells were also negative for *Pdgfra*-H2BGFP (Figure 4E).

Thus, our data fail to support EndoMT or epithelial-to-mesenchymal transition (EMT) as mechanisms for tubulointerstitial fibrosis in the UUO model, indicating that deficient TIE2 signaling does not promote EndoMT in this context.

### Pharmacological or genetic TIE2 activation prevents UUO-induced tubular injury and PDGFB expression.

**Figure 5:**
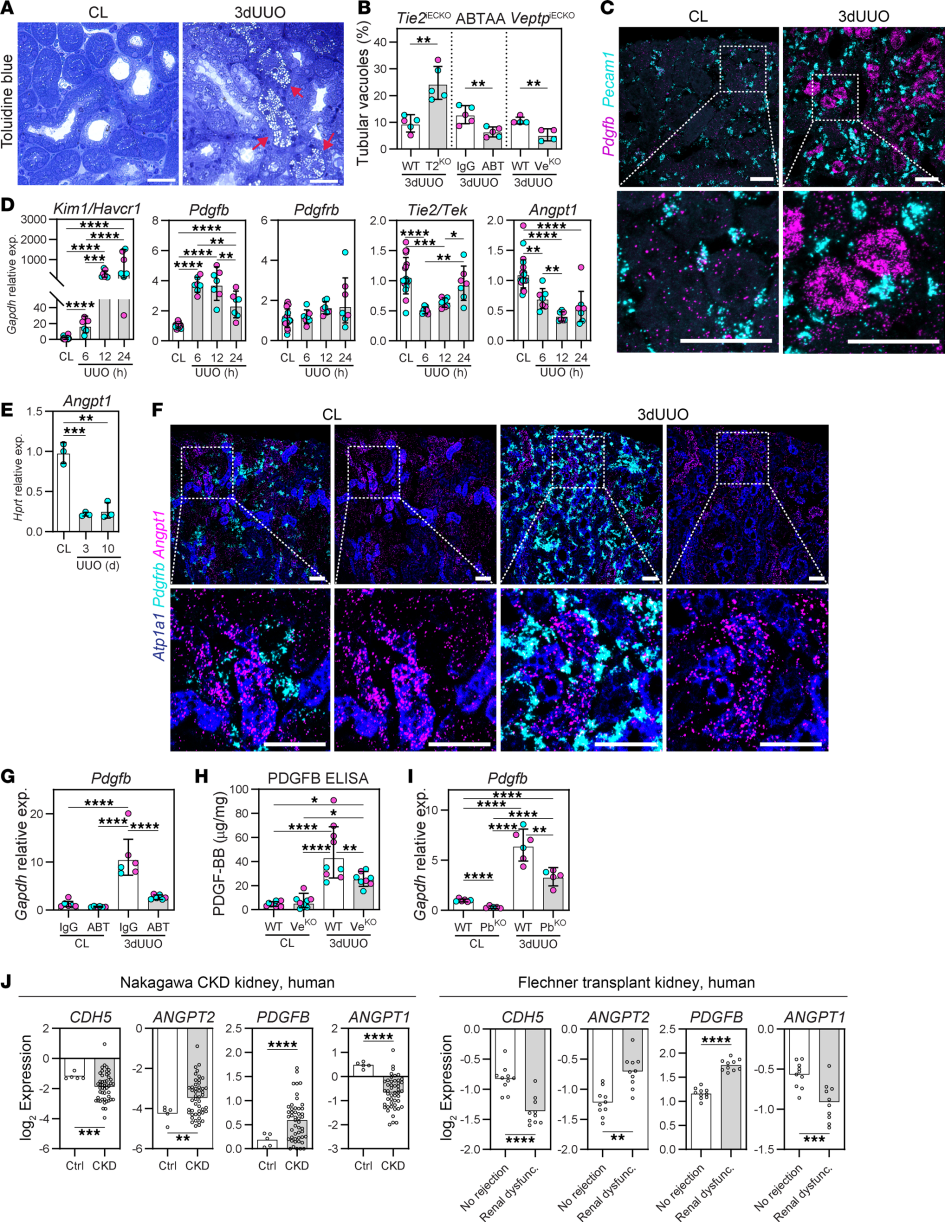
Pharmacological or genetic TIE2 activation prevents UUO-induced tubular injury and PDGFB expression. (**A**) Tubular segments with pathological vacuoles (red arrow) in kidney sections stained with toluidine blue. (**B**) Quantification of tubular segments with vacuoles from ABTAA-treated (ABT), *Veptp*^iECKO^ (Ve^KO^), and *Tie2*^iECKO^ (T2^KO^) mice. Data are based on *n* = 4–5 mice/group and >10,000 tubular cross sections. (**C**) RNA-ISH for *Pecam1* (cyan) and *Pdgfb* (magenta) in 3-day UUO kidneys. Representative image of *n* = 4 mice. Scale bars: 50 μm. (**D** and **E**) Gene expression of *Kim1*, *Pdgfb*, *Pdgfrb*, *Tie2*, and *Angpt1* in UUO kidneys from indicated time points. Data are based on *n* = 3–7 mice/group. (**F**) RNA-ISH for *Angpt1* (magenta), mesenchymal marker *Pdgfrb* (cyan), and tubular marker *Atp1a1* (blue) in 3-day UUO kidneys. Representative image of *n* = 3 mice. Scale bars: 50 μm. (**G**–**I**) Expression of Pdgfb/PDGFB in 3-day UUO kidneys from ABTAA-treated, *Veptp*^iECKO^, and *Pdgfb*^iKO^ (Pb^KO^) mice. Data are based on *n* = 5–9 mice/group. (**J**) Patient data retrieved from Nephroseq for renal *CDH5* (endothelial marker), *ANGPT2*, *PDGFB*, and *ANGPT1* expression in CKD and renal dysfunction compared with normal human kidney. Data in graphs (**B**, **D**, **E**, and **G**–**I**) represent mean ± SD, and each symbol represents 1 mouse (females, magenta; males, cyan). *P < 0.05, **P < 0.01, ***P < 0.001, and ****P < 0.0001, determined by 1-way ANOVA. Human data (**J**) represent Log2 expression and statistical differences from Nephroseq (see Methods).

The renal tubular system is densely packed with mitochondria and relies on oxidative phosphorylation, making it vulnerable to ischemic injury (65). We aimed to analyze whether TIE2 activation in the vasculature could protect the tubular system in our CKD model. UUO-induced tubular vacuoles were assessed as an indicator of tubular injury (for details, see Methods) (Figure 5A). Cortical tubular segments exhibiting UUO-induced pathological vacuoles were significantly reduced in ABTAA-treated and *Veptp*^iECKO^ mice compared with IgG-treated and WT controls (Figure 5B). In contrast, *Tie2*^iECKO^ mice showed substantially greater tubular injury compared with WT mice (Figure 5B).

PDGFB is a known mitogen for perivascular mesenchymal cells and is upregulated in kidney diseases (66). While TIE2 has been shown to regulate *Pdgfb* in endothelial cells (67, 68), our RNA-ISH analysis revealed that *Pdgfb* expression was significantly upregulated in tubular segments following UUO, with no effect on isolated endothelial cells (Figure 5C and Supplemental Figure 6E). This finding, along with recent single-cell RNA-Seq data in experimental CKD (47, 69), support the idea of using *Pdgfb* as a marker for tubular injury.

To further investigate the early regulation of injury markers, we performed gene expression analysis at 6, 12, and 24 h after UUO. We observed a notable and sustained increase in kidney injury molecule 1 (*Kim1/Havcr1*) and *Pdgfb*, beginning at 6 h after UUO, indicating early tubular injury (Figure 5D). At these early time points, mesenchymal activation had not yet occurred, as indicated by the stable *Pdgfrb* expression throughout the analysis. UUO also led to reduced expression of both *Tie2*/*Tek* and *Angpt1* (Figure 5D). *Angpt1* expression was also suppressed 3 and 10 days after UUO (Figure 5E), which we have previously shown (5). RNA-ISH demonstrated that *Angpt1* was expressed in both mesenchymal cells and tubular epithelial cells, with UUO-induced reductions primarily affecting tubular cells (Figure 5F).

Importantly, UUO-induced expression of *Pdgfb*/PDGFB was significantly prevented in ABTAA-treated and *Veptp*^iECKO^ mice (Figure 5, G and H), supporting the notion of vascular-mediated protection of the tubular system. As expected, *Pdgfb*^iKO^ mice exhibited reduced *Pdgfb* expression in both UUO and contralateral (unobstructed) kidneys (Figure 5I). Our data support a sequence of events whereby vascular integrity regulates tubular injury and PDGFB expression, which in turn influences the degree of tubulointerstitial fibrosis. Notably, patients with CKD or renal dysfunction display changes in corresponding genes, such as a loss of the endothelial marker CDH5, an increase in *ANGPT2* and *PDGFB*, and a reduction in *ANGPT1* (Figure 5J).

### Post-injury treatment with ABTAA slows progression of fibrosis.

**Figure 6:**
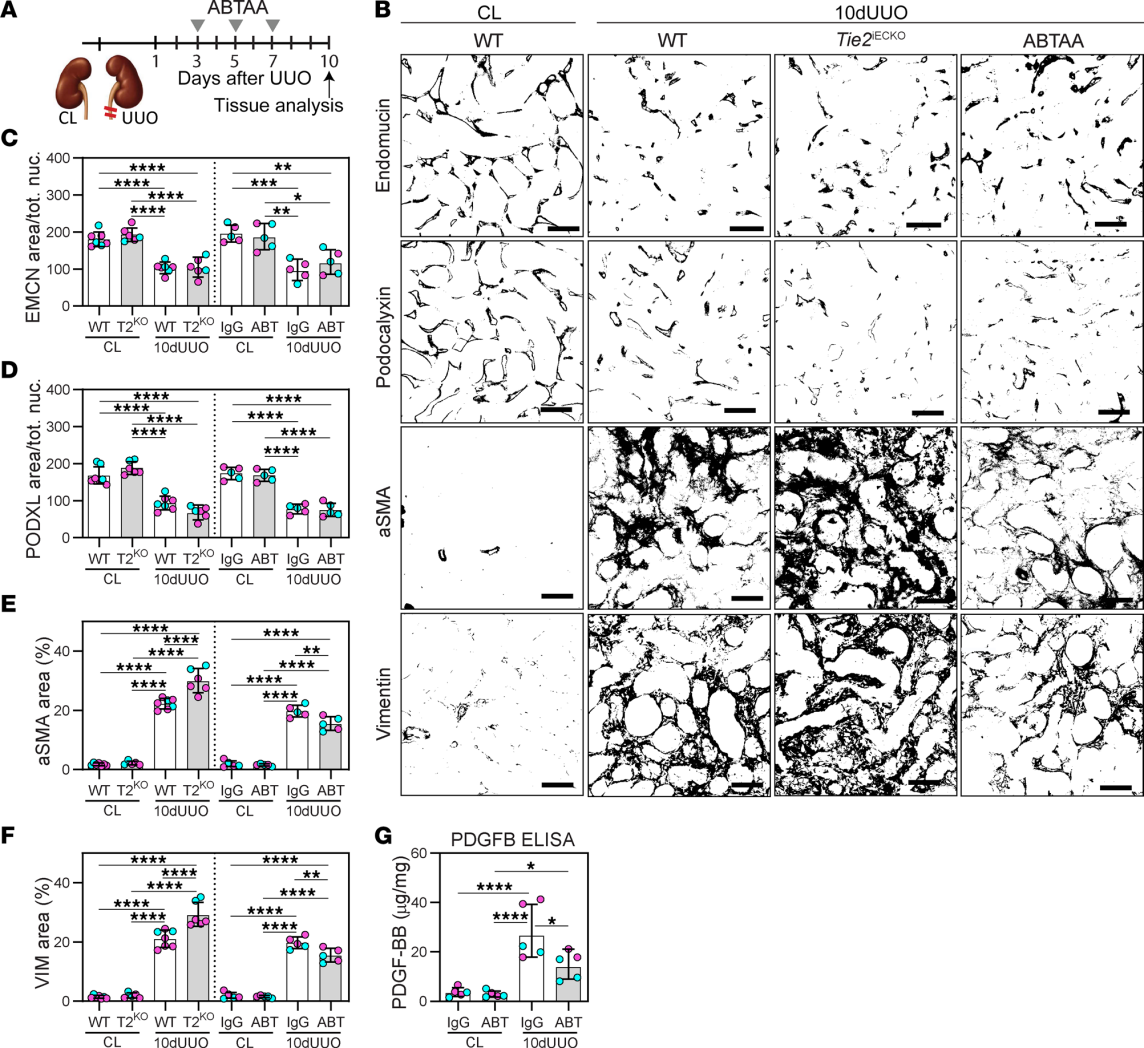
Post-injury treatment with ABTAA slows progression of fibrosis. (**A**) Schematic diagram of administration of ABTAA for evaluation in 10-day UUO kidneys. (**B**–**F**) Immunohistochemistry of renal cortex for capillary density (endomucin [EMCN] and podocalyxin [PODXL]) and tubulointerstitial fibrosis (aSMA and vimentin) in 10-day UUO kidneys from ABTAA-treated mice (ABT) and *Tie2*^iECKO^ (T2^KO^) mice. Data are based on *n* = 6–7 mice/group and quantification of >220 images/marker. Scale bars: 50 μm. (**E**) Protein concentration for PDGFB in 10-day UUO kidneys from ABTAA-treated mice. Data are based on *n* = 5 mice/group. Data in graphs represent mean ± SD, and each symbol represents 1 mouse (females, magenta; males, cyan). *P < 0.05, **P < 0.01, ***P < 0.001, and ****P < 0.0001, determined by 1-way ANOVA and Tukey’s post hoc test.

To further investigate the therapeutic potential of ABTAA treatment, we implemented a more clinically relevant treatment regimen. Specifically, we initiated ABTAA treatment 3 days after UUO injury and assessed the kidneys at day 10 (Figure 6A). In this context, UUO-induced capillary density in the ABTAA-treated mice was similar to that observed in the IgG-treated mice (Figure 6, B–D). However, post-injury ABTAA treatment significantly reduced UUO-induced tubulointerstitial fibrosis, as indicated by aSMA and vimentin staining, while *Tie2*^iECKO^ mice exhibited a significant increase in fibrosis (Figure 6, E and F). Additionally, tubular injury, as reflected by PDGFB expression, was notably prevented in ABTAA-treated mice, suggesting improved vascular function, although this may not be due to preserved capillary density (Figure 6G).

### Pharmacological or genetic TIE2 activation, or inhibition of PDGFB signaling, protects the kidney transcriptome following UUO injury.

**Figure 7:**
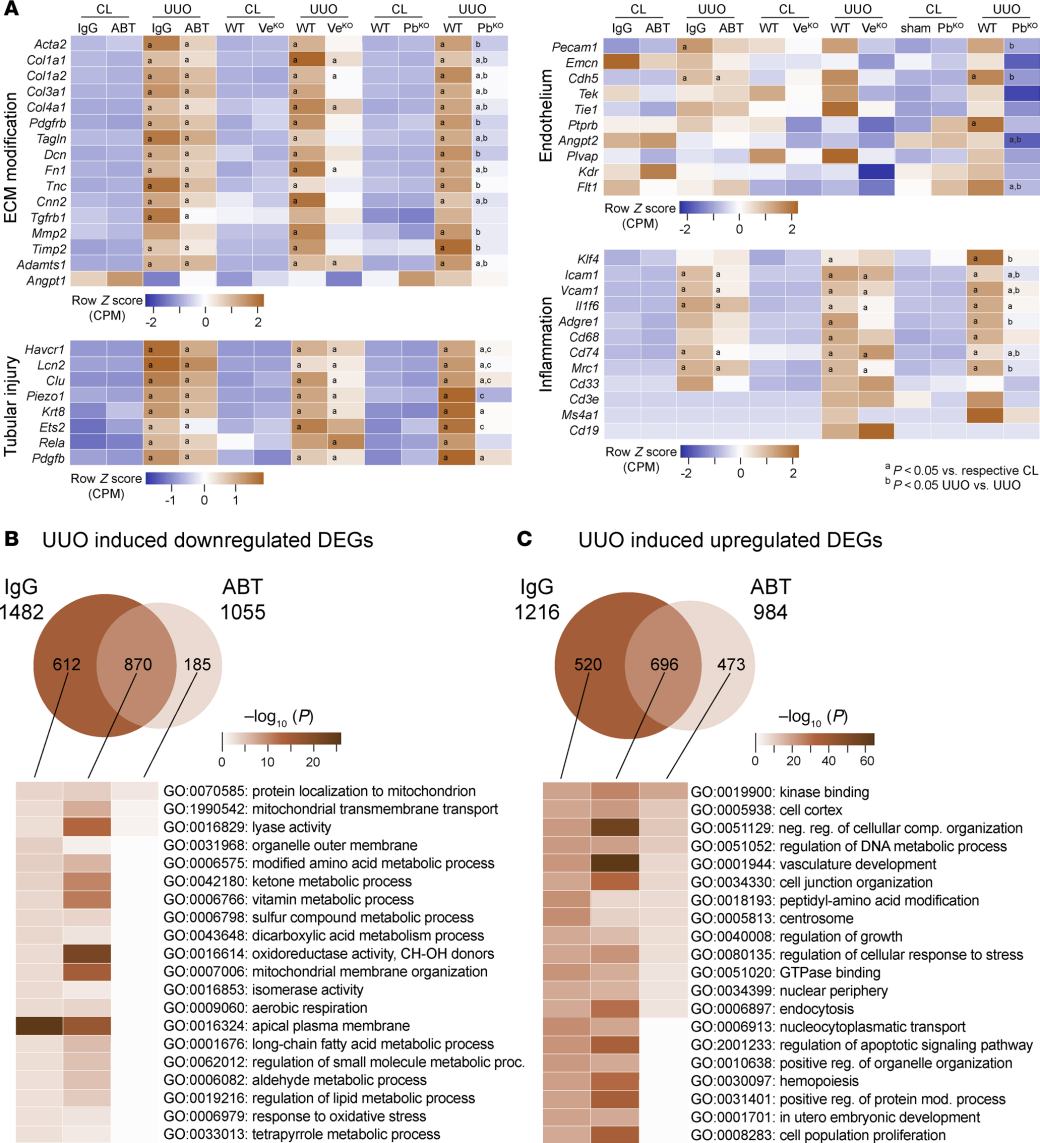
Pharmacological or genetic TIE2 activation, or inhibition of PDGFB signaling, protects the kidney transcriptome following UUO injury. Bulk RNA-Seq of 3-day UUO kidneys. (**A**) Heatmaps of genes relevant for ECM modifications, tubular injury, endothelium, and inflammation in ABTAA/IgG-treated (*n* = 7–8), *Veptp*^iECKO^/*Veptp*^WT^ (*n* = 6–8), and *Pdgfb*^iKO^/*Pdgfb*^WT^ (*n* = 4) mice. ^a^*P* < 0.05 versus respective CL; ^b,c^*P* < 0.05 UUO versus UUO. (**B** and **C**) Venn diagrams of downregulated and upregulated DEGs and Metascape analysis of linked GO for DEGs.

Bulk RNA-Seq of whole kidneys was used to investigate transcriptional changes in ABTAA-treated, *Veptp*^iECKO^, and *Pdgfb*^iKO^ mice 3 days after UUO. We visualized genes associated with ECM modification, tubular injury, endothelial function, and inflammation in heatmaps across our experimental groups (Figure 7A and Supplemental Data File 1). As expected, UUO-induced ECM modification genes, including *Acta2*, *Col1a1*, *Col1a2*, *Col3a1*, *Col4a1*, *Pdgfrb*, *Fn1*, *Tagln*, *Dcn*, *Cnn2*, *Tnc*, *Mmp2*, *Timp2*, and *Adamts1*, along with tubular injury markers, such as *Havcr1*, *Lcn2*, *Clu*, *Piezo1*, *Krt8*, *Ets2*, *Rela*, and *Pdgfb*, were significantly upregulated compared with the contralateral kidneys in their respective groups. Both ABTAA treatment and *Veptp*^iECKO^ elicited a generally reduced response, though most did not reach statistical significance (Figure 7A and Supplemental Data File 2). Notably, *Pdgfb*^iKO^ demonstrated significant protection against most ECM modification and tubular injury genes. Only a few of the endothelial genes (*Pecam1*, *Emcn*, *Cdh5*, *Tek*, *Tie1*, *Ptprb*, *Angpt2*, *Plvap*, *Kdr*, and *Flt1*) were significantly regulated (Figure 7A), despite the pronounced capillary rarefaction and loss of fenestrations documented in experimental data (Figures 1 and 2). The Discussion section addresses potential reasons for these observations. UUO-induced endothelial activation markers (*Klf4*, *Icam1*, and *Vcam1*) were present in several injured kidneys but were partly blunted in ABTAA-treated, *Veptp*^iECKO^, and *Pdgfb*^iKO^ mice without reaching statistical significance in most cases (Figure 7A and Supplemental Data File 2). Overall, the inflammatory response was relatively low (see Discussion), although an increase in UUO-induced macrophage markers (*Adgre1*, *Cd68*, *Mrc1*, and *Ccr2*) was observed in *Veptp*^iECKO^ and *Pdgfb*^iKO^ mice, unlike markers for neutrophils (*Cd33*), T cells (*Cd3e*), or B cells (*Ms4a1* and *Cd19*) (Figure 7A). A searchable database for all genes is available: https://heomics.shinyapps.io/Jeansson_lab_kidney_UUO/

We subsequently conducted a more in-depth analysis of differentially expressed genes (DEGs) in ABTAA-treated animals compared with controls treated with IgG (Figure 7, B and C). In IgG-treated mice, UUO-induced kidney injury resulted in downregulation of 1,482 genes and upregulation of 1,216 genes compared with contralateral kidneys. In contrast, ABTAA treatment exhibited a blunted response, with 1,055 downregulated and 984 upregulated DEGs (Figure 7, B and C, and Supplemental Data Files 2 and 3). To elucidate the differences in transcriptional programs, we performed Gene Ontology (GO) enrichment analysis for unique and common UUO-induced DEGs from both ABTAA- and IgG-treated groups using Metascape (70). The downregulated unique DEGs in the IgG group and the common DEGs between IgG and ABTAA displayed significant alterations in pathways related to metabolic processes and oxidative phosphorylation, likely reflecting tubular injury due to the high metabolic activity and high mitochondrial density of tubular epithelial cells (71). In comparison, the DEGs specific to ABTAA treatment showed a diminished or absent response in terms of metabolic processes and oxidative phosphorylation (Figure 7B and Supplemental Data File 4).

In contrast, many of the UUO-induced common upregulated DEGs (from both IgG and ABTAA) were associated with response to wounding, ECM, cell adhesion, and collagen and integrin binding (Figure 7C and Supplemental Data File 4). These pathways were in many cases also significant in IgG-treated animals. Interestingly, ABTAA treatment appeared to partly blunt these effects (Figure 7C and Supplemental Data File 4).

## Discussion

Our study presents evidence that vascular protection through TIE2 activation via ABTAA is a promising novel therapeutic strategy for CKD. ABTAA’s unique ability to bind and cluster ANGPT2 into a potent TIE2 activator helps to prevent endothelial dysfunction, tubular injury, and tubulointerstitial fibrosis in experimental CKD. Findings from genetically modified mice supported this conclusion; TIE2 activation resulting from endothelial *Veptp*-knockout provided a similar benefit, while endothelial *Tie2*-knockout mice exhibited worse kidney damage. Our experiments underscore the critical role of vascular protection in maintaining tubular health, as failure in this process leads to increased profibrotic PDGFB expression and subsequent tubulointerstitial fibrosis.

Previous studies have shown that ABTAA normalizes blood vessels in tumors (40), mitigates sepsis and sepsis-induced injury (39), and promotes choriocapillary regeneration in macular degeneration (41). Notably, ABTAA offered greater protection in sepsis compared with ANGPT2 inhibition alone (39), highlighting its dual beneficial effects: (a) the removal of ANGPT2’s antagonistic activity on TIE2 and (b) the enhanced TIE2 signaling through the conversion of ANGPT2 into a higher-order oligomer that effectively activates TIE2 (39). Our current study illustrates that several modifiers of TIE2 signaling, aside from ANGPT2, are dysregulated in experimental CKD. We observed UUO-induced VEPTP expression in the kidney, consistent with previous reports in models of diabetic nephropathy, ischemia/reperfusion injury, and HIF2α overexpression (34, 35). Additionally, *Angpt1* decreased within 6 h after UUO, shown previously to be expressed by both tubular epithelial cells and perivascular mesenchymal cells (72–74). Although *Angpt1* expression is highest in mesenchymal cells, their relatively low number compared with the epithelial cells suggests that both sources contribute to biologically relevant levels of ANGPT1 in the kidney. RNA-ISH performed 3 days after UUO in this study indicated reduced *Angpt1* expression primarily in tubular epithelial cells. Recent single-cell RNA-Seq data after ischemia/reperfusion injury and UUO have confirmed the reduction of *Angpt1* transcripts in injured or repairing proximal tubular cells, as well as the loss of *Angpt1* in activated mesenchymal cells (myofibroblasts) (73).

From a therapeutic perspective, ABTAA offers several advantages compared with other TIE2-activating strategies. ABTAA activates TIE2 only in the presence of ANGPT2, as demonstrated by Han et al. (39). This was also confirmed in the current study; systemic administration of ABTAA selectively restored TIE2 phosphorylation in injured kidneys with elevated ANGPT2 levels, without activating TIE2 in healthy, vascular-rich lung tissue. This finding supports the injury-targeting mechanism of ABTAA and reduces concern for vascular enlargement and venous malformation, previously demonstrated to result from sustained TIE2 overactivation and *Veptp* deletion (35, 75–77). Importantly, VEPTP does not solely dephosphorylate TIE2, and VEPTP targeting may therefore also affect the phosphorylation of other proteins (33, 78). While ABTAA treatment initiated after UUO injury effectively prevents fibrosis, the PTC density was not preserved. Although the vessels may remain functionally improved with late-onset ABTAA treatment, these findings suggest that once capillary integrity and density are compromised, restoration may be challenging, particularly in the rapidly progressing UUO model. Other studies suggest that capillary injury precedes fibrosis in various preclinical models, including UUO, ischemia/reperfusion, and Alport syndrome (17, 73). This is supported by our current study, where experiments from 6 to 24 h after UUO revealed effects on vascular and tubular injury markers prior to detectable mesenchymal cell activation.

Our results demonstrate that kidney injury leads to increased tubular PDGFB expression in CKD, a finding also identified in recent single-cell RNA-Seq studies of experimental CKD (47, 69, 73). Importantly, this study shows that vascular TIE2 activation can attenuate increased PDGFB levels. Additionally, we found that knocking out *Pdgfb* reduces tubulointerstitial fibrosis, confirming the significance of this pathway in mesenchymal cell activation and fibrosis, as previously reported (49–56, 79). However, while PDGFB is known to regulate fibrosis, it is noteworthy that *Pdgfb*-knockout does not protect against loss of capillary density. Therefore, targeting PDGFB may not be a viable therapeutic approach, especially given that *Pdgfb*-knockout leads to a progressive loss of capillary pericyte coverage (48). The roles of EndoMT and EMT in renal fibrosis have been widely debated (63, 80, 81). Given TIE2’s importance as an endothelial signaling factor, we investigated whether loss of *Tie2* could promote EndoMT in our experimental CKD model. For this purpose, we utilized a *Cdh5*-TdTomato lineage tracer alongside a *Pdgfra*-H2BGFP reporter to identify putative endothelial cells transitioning toward a mesenchymal phenotype. This approach revealed a UUO-induced increase in mesenchymal cells but provided no evidence of EndoMT or EMT contributing to this process.

Several molecular mechanisms have been proposed to explain the beneficial effects of TIE2 activation in providing vascular protection. TIE2 activation triggers a cascade of downstream signaling events that lead to nuclear exclusion of FOXO1, which in turn increases nitric oxide synthase and local NO production, a pathway previously shown to be renoprotective in CKD models (34, 82, 83). Inflammation and macrophage infiltration are integral to the progression of renal injury in UUO (84) and CKD in general (85). TIE2-activating compounds, including ABTAA and ANGPT2 inhibitors, are known to exhibit antiinflammatory effects (27, 39). In our current study, the ethical requirement for administrating UUO mice with an analgesic/antiinflammatory drug potentially dampened the inflammatory response, which may explain the minimal injury-induced inflammation observed. Our study confirmed the occurrence of capillary rarefaction, and it showed that PTC perfusion and fenestrations were affected by disease progression, in line with findings from Babickova et al. (16). The energy demands of tubular epithelial cells, particularly proximal tubular epithelial cells, are high, and these cells rely heavily on the functionality of nearby PTCs for their aerobic metabolism. The UUO-induced downregulation of DEGs linked to metabolic processes and oxidative phosphorylation observed in this study likely reflects injury to tubular epithelial cells, as they represent the majority of cells in the kidney.

While our study was limited to a single experimental model of CKD, the regulation of TIE2 signaling appears to be consistent across various studies and experimental models. ANGPT1, ANGPT1 mimetics, modified ANGPT1, genetic overexpression of *Angpt1*, *Veptp*-knockout, and ANGPT2 inhibition have all demonstrated protective effects in preclinical models of kidney injury, including UUO, ischemia/reperfusion, and conditions induced by diabetes (including *db*/*db*, streptozotocin-induced, and *Akita* mice), cyclosporine, and sepsis (23, 25–27, 35–38). Our bulk RNA-Seq data for individual genes showed trends matching other experimental findings within our study regarding ECM regulation and tubular injury, but statistically significant differences were not reached for most transcripts. Neither capillary rarefaction nor loss of markers of fenestration was observed in our RNA-Seq data. While posttranscriptional mechanisms and protein half-life can contribute to discrepancies between mRNA and protein data, the likely explanation in this case is that the experiment was underpowered (*n* = 7–8/group). Variability was high in the groups due to treatment effects or gene knockout, compounded by the kidney injury model. It is also possible that the use of whole kidney tissue in RNA-Seq did not accurately reflect changes seen in the cortex.

In conclusion, our data demonstrate that CKD progression is regulated by TIE2 signaling and endothelial dysfunction. We further show that ABTAA acts locally in conjunction with increased ANGPT2 levels to protect the renal endothelium, which in turn preserves tubular health, downregulates tubular PDGFB expression, and limits tubulointerstitial fibrosis. These findings provide mechanistic insights into kidney disease progression and offer a strong rational for further testing of ABTAA as a treatment of kidney disease in clinical trials.

## Methods

### Sex as a biological variable.

Our study examined male and female animals, and similar findings are reported for both sexes. Sex as a biological variable was not the subject of the study, and some groups have an imbalanced sex distribution; therefore, we report the sex in each graph (females, magenta; males, cyan) but do not draw any conclusions about sex differences in the study.

### Study design.

The major objective of this study was to characterize TIE2 signaling in progression of CKD and to investigate the therapeutic value of TIE2 activation. Mice were subjected to UUO as an experimental model of CKD. Kidney injury was evaluated up to 10 days after injury. Treatment with ABTAA was used as a pharmacological approach to activate TIE2 and reduce kidney injury after UUO. Similarly, Cre-dependent endothelium-specific knockout of *Veptp* was used to genetically increase TIE2 activation. Conditional endothelial knockout of *Tie2* served as a TIE2-incompetent signaling model. The causal effect of PDGFB on tubulointerstitial fibrosis was investigated with conditional global knockout of *Pdgfb*.

### Animals.

Floxed *Veptp* (34) and *Tie2* (86, 87) mice were crossed with tamoxifen-inducible *Cdh5*-Cre^ERT2^ mice (88) to generate endothelium-specific knockout of the genes (*Veptp*^iECKO^ and *Tie2*^iECKO^, respectively). *Tie2* mice were also crossed with a reporter mouse, Ai14-TdTomato [Gt(ROSA)26Sor^tm14(CAG-tdTomato)Hze^; The Jackson Laboratory; 007914] (89), resulting in a lineage tag of endothelial cells. Littermate controls were tamoxifen-induced *Tie2* wt/wt *Cdh5*-Cre^ERT2^ mice for the *Tie2*-knockouts and noninduced *Veptp* wt/lox *Cdh5*-Cre^ERT2^ for *Veptp-*knockouts. Floxed *Pdgfb* mice (Pdgfb^tm2Cbet^) (90) were crossed with tamoxifen-inducible *Actb*-Cre^ERT2^ mice [Tg(CAG-cre/Esr1*)5Amc; The Jackson Laboratory; 004682] (91) to generate whole-body knockout of *Pdgfb* (*Pdgfb*^iKO^). Littermate controls were Cre^–^ *Pdgfb* wt/lox or lox/lox mice. Recombination was induced with 3 doses of tamoxifen (2 mg) in peanut oil by oral gavage at 4 weeks of age (*Tie2*^iECKO^ and *Veptp*^iECKO^) or 1 week prior to experiments (*Pdgfb*^iKO^). For some experiments, additional crossings were done to obtain a reporter for myofibroblasts with *Pdgfra*-H2BGFP [Pdgfra^tm11(EGFP)Sor^] (64). Mice were on a mixed background. Mice were genotyped with the primers described in Supplemental Table 1. ABTAA has been described previously (39, 40) and was a gift from Gou Young Koh (IBS Center for Vascular Research, Daejeon, South Korea). C57BL6/J mice from in-house breeding or WT mice from the above-mentioned breeding were used for treatment with ABTAA and for early gene regulation analysis. Mice were injected i.p. with ABTAA (25 mg/kg body weight in PBS) at indicated time points in Figure 1A and Figure 6A. Control mice were injected with the same dose of human IgG Fc (AG714; Millipore) in PBS. Adult mice (8–16 weeks) were used for all experiments. Animals were housed on a 12 h light–dark cycle with ad libitum access to water and standard chow. CKD was induced by UUO as previously described (5). UUO surgery was performed under anesthesia, and animals received analgesia (Carprofen 5 mg/kg s.c.; Norocarp; N-vet AB) administered before surgery and daily 2 days after surgery. Sham mice were subjected to all procedures except ligation of the ureter. Sham mice were used as baseline controls for left/right kidney perfusion in contrast-enhanced ultrasound imaging (Figure 1H). Both female and male mice were used in all experiments as the UUO model has previously not shown any gender differences (92). The UUO model was chosen due to its reproducibility and inclusion of key features of human CKD, including PTC loss, tubulointerstitial fibrosis, and increased ANGPT2 expression. The contralateral kidney served as an internal control, allowing for reduction in animal numbers.

The total number of mice, gender distribution, and weight distribution can be found in Supplemental Table 2. All experiments used at least 2 independent litters, and a single animal was 1 experimental unit. The sample size and end points were selected based on our previous studies with the experimental model (5). No criteria for including or excluding animals were set, and no data were excluded or defined as outliers. No randomization of treatment was used. Blinding was performed at different stages of the experiments. Animal care staff were unaware of treatment groups. Persons performing experimental measurements were blinded until grouped data analysis.

### Renal perfusion measured with contrast imaging ultrasound.

Renal perfusion was measured in isoflurane-anesthetized mice on a heated platform utilizing a Vevo 2100 ultrasound system with a MS250 transducer and contrast imaging functionality software (Visual Sonics; Fujifilm). Mice were kept at a body temperature of 36°C–37°C, continuously measured with an anal probe. Mice were imaged from the back, and the ultrasound transducer was fixed in place with a mechanical positioning system. Regular B-mode images were used to optimally position the mice to enable imaging of both kidneys at the same time. Mice were tail vein injected with 100 μL microbubble-based contrast agent (Vevo MicroMarker; VS-11913; Fujifilm Visual Sonics) from vials resuspended with 2 mL saline. Nonlinear contrast images were acquired with general imaging in the Vascular Package (Fujifilm Visual Sonics ) at 21 GHz. Images were analyzed for contrast intensity with Vevo Lab 3.2.6 (Fujifilm Visual Sonics) after manually marking the region of interest of cortex of both contralateral control (CL**)** and UUO kidneys (Supplemental Figure 7). Perfusion was normalized to CL kidneys for each mouse. Five sham-operated WT mice were used in ultrasound graphs and presented as the left kidney normalized to the right kidney for comparison.

### Immunohistochemistry.

Mice were euthanized with cervical dislocation; kidneys were dissected and renal capsules removed. Pieces of CL and UUO kidney were fixed for 4 h at room temperature with 4% buffered formaldehyde (Histolab Products AB) and washed with PBS followed by vibratome sectioning of 100 μm thick sections. After blocking and permeabilization (X0909; Dako; with 0.25% Triton X-100 for 2 h), sections were incubated with primary antibodies overnight at 4°C on a shaker. Antibodies are described in Supplemental Table 3. Appropriate secondary antibodies were added after washing with PBS containing 0.05% Tween 20 and incubated for 2 h at room temperature. Nuclei were stained with Hoechst 33342 (Thermo Fisher Scientific).

### RNA-ISH.

The RNAscope Multiplex Fluorescent Reagent Kit (v.2) (ACDBio) and TSA Plus reagents (PerkinElmer) were used according to the manufacturers’ protocol for fresh frozen or fixed frozen sections with minor modifications. In brief, frozen tissue sections were cut at 14 μm thickness. Fresh frozen sections were first fixed with 4% buffered formaldehyde (Histolab Products AB) for 20 minutes at 4°C, washed twice with 1× PBS, and dehydrated through an ethanol series. Fixed frozen sections were ready directly for dehydration. After dehydration, HRP was quenched with Bloxall blocking solution (Sp-6000; Vector Technologies) for 10 min at room temperature followed by Pretreat III solution (ACDBio) for 30 min at room temperature. RNAscope probes (ACDBio) were hybridized on the sections for 2 h at 40°C, after which fluorescence signals were developed and amplified according to the manufacturer’s protocol. Sections were mounted with ProLong Gold mounting medium (Thermo Fischer Scientific) and images obtained using a confocal microscope (SP8; Leica Microsystems). RNAscope probes used were as follows: *Pecam1* (catalog 316721), *Pdgfb* (catalog 424651), *Angpt1* (catalog 449271), *Pdgfrb* (catalog 411381), *Pdgfra* (catalog 480661), and *Atp1a1* (catalog 569611).

### Proliferation assay (EdU).

For EdU experiments, 0.25 mg EdU was injected i.p. at indicated time points, as shown in Supplemental Figure 5. Visualization of EdU^+^ cells was done with a Click-it plus EdU Imaging kit (C10640; Thermo Fisher Scientific) on 100 μm vibratome sections following the manufacturer’s instructions.

### Quantitative real-time PCR.

RNAeasy Micro or Mini kit (Qiagen) was used to extract mRNA according to the manufacturer’s protocol, followed by cDNA synthesis of 1 μg mRNA using iScript reverse transcription Supermix (170-8841; Bio-Rad). Real-time PCR was performed using cDNA with TaqMan Gene Expression Master Mix (4369016; Thermo Fisher Scientific) together with probes on a CFX-96 real-time PCR system (Bio-Rad). For primers, iTaq Universal SybrGreen Supermix was used (172-5130; Bio-Rad). Probes and primers are listed in Supplemental Table 4. Expression results were normalized to endogenous control *Hprt* or *Gapdh*, and relative quantification was done using the Livak method (2^–ΔΔCT^) (93). FACS-sorted cells were subjected to a preamplification step of 12 cycles with SsoAdvanced PreAmp Supermix (172-5160; Bio-Rad) according to the manufacturer’s instructions.

### FACS of endothelial cells.

Endothelial cells were isolated from CL and UUO kidneys of WT and *Tie2*^iECKO^ mice utilizing the *Cdh5*-TdTomato lineage labeling. At dissection, kidneys were cut into small pieces and digested into a single-cell suspension in gentleMACS C tubes (Miltenyi Biotec) with DMEM containing 0.13 U/mL Liberase TL (5401020001; Roche, Merck) and 1 μL/mL DNase I (18068-15; Invitrogen) with rotation (200 rpm) in a gentleMACS dissociator (Miltenyi Biotec) for 20 minutes at 38°C. The suspension was passed through a 100 μm cell strainer, and cells were washed 2 times by centrifugation at 300*g* for 5 min in DMEM containing 5 mM EDTA and 1% FBS. To reduce epithelial cell content from the cell suspension, we utilized CD10-conjugated Dynabeads as previously described (47). Beads were prepared by adding 20 μL/mL rabbit monoclonal CD10 antibody to the Protein G Dynabeads (10004D; Thermo Fisher Scientific) for 10 minutes at room temperature. After washing, CD10-Dynabeads were incubated with cell suspension for 10 minutes at room temperature, and cell solution remaining after magnet removal of CD10-Dynabead–bound cells was used for *Cdh5*-TdTomato isolation by the BD FACSMelody cell sorter (BD Biosciences). Cells were sorted directly into RLT lysis buffer (Qiagen), and RNA was purified as above.

### Protein analysis.

Snap-frozen kidney tissues were homogenized in RIPA buffer (89900; Pierce) containing protease and phosphatase inhibitors (4693116001 and 4906845001; Merck). After centrifugation, the supernatant was collected and measured for protein concentration using a bicinchoninic acid (BCA) assay (Pierce), aliquoted, and stored at –80°C. Protein lysates were used to measure protein concentrations by ELISA for TIE2 (MTE200; R&D Systems), ANGPT2 (MANG20; R&D Systems), and PDGFB (ab224879; Abcam) according to the manufacturers’ instructions.

For Western blotting, proteins were separated under reducing conditions on 4%–12% Criterion XT Bis-Tris gels (3450123; Bio-Rad) and then transferred using Criterion wet transfer to 0.2 μm PVDF membranes (1620239; Bio-Rad). Blots were blocked with EveryBlot blocking buffer (Bio-Rad) and incubated overnight at 4°C with primary antibody (Supplemental Table 3). After washing and incubation with the appropriate HRP-conjugated secondary antibody, proteins were visualized using SuperSignal West Femto detection reagent (34096X4; Thermo Fisher Scientific). Membranes were cut to probe for different sized proteins. Band density was quantified with ImageJ (NIH).

For TIE2 immunoprecipitation, snap-frozen kidney tissues were homogenized in ice-cold IP lysis buffer (Pierce 87787) with 2× protease and 1× phosphatase inhibitors, as listed above, and 1 mM Na_3_VO_4_ and incubated for 2 h at 4°C in rotation. After centrifugation and BCA assay for protein concentration analysis, 2.5 mg of total kidney lysate or 1.5 mg lung lysate per sample was incubated with rabbit anti-TIE2 antibody (19157-1-AP; Thermo Fischer Scientific) overnight rotating at 4°C. The following day, lysates were incubated with Protein-A agarose beads (9863; Cell Signaling) for 2 h rotating at 4°C and subsequently washed 4 times with lysis buffer and denatured in 2× NuPage LDS sample buffer with denaturing agent (NP0007; Life Technologies) at 97°C for 5 min. Proteins were separated under reducing conditions on 4%–12% Bis-Tris gels (NuPage; Invitrogen) and transferred to 0.4 μm PVDF membranes. Membranes were blocked with 3% BSA and TBS + 0.1% Tween 20 (TBST) and incubated for 2 or 3 nights at 4°C with primary antibody (4G10; 05-321; Merck). After washing with TBST and incubation with appropriate HRP-conjugated secondary Ab (in BSA-TBST), membranes were developed with ECL Prime Western Blotting Detection Reagent (10308449; Cytiva Amersham). The membranes were stripped and reprobed with goat anti-mouse TIE2 Ab (AF762; R&D Systems). All antibodies are listed in Supplemental Table 3.

Blood samples for plasma ANGPT2 measurements were collected by heart puncture in isoflurane-anesthetized mice and collected in tubes with K2 EDTA (Microvette 20.1339.100; Sarstedt). Samples were centrifuged at 2,500*g* for 10 minutes, and plasma was collected and stored at –80°C until analysis. ANGPT2 in plasma was measured by ELISA (MANG20; R&D Systems) in duplicates according to the manufacturer’s instructions.

### Transmission electron microscopy.

For electron microscopy, kidneys were cut in 1 mm cubes, immersion fixed in 2.5% glutaraldehyde (Ted Pella) and 1% paraformaldehyde (Merck) in 0.1 M phosphate buffer, pH 7.4, and stored at 4°C until further processing. Samples were rinsed in 0.1 M phosphate buffer for 10 min followed by 1 h incubation in 1% osmium tetroxide (TAAB Laboratories Equipment) in 0.1 M phosphate buffer. After rinsing in phosphate buffer, samples were dehydrated in alcohol followed by 5 min of incubation in propylene oxide (TAAB Laboratories Equipment). Samples were then incubated for 1 h in Epon Resin (TAAB Laboratories Equipment) and propylene oxide (1:1), followed by overnight incubation in 100% resin. Subsequently, samples were embedded in capsules with fresh resin for 1–2 h and then heat cured at 60°C for 48 h. The specimens were cut into semithin sections, stained with toluidine blue, and examined by light microscopy. Ultrathin sections (60–70 nm) were cut in an EM UC7 Ultramicrotome (Leica) and placed on grids. Sections were contrasted with 5% uranyl acetate and Reynold’s lead citrate and visualized in a Tecnai G2 Spirit BioTwin electron microscope (Thermo Fisher Scientific/FEI) at 80 kV with an Orius SC200 CCD camera and Gatan Digital Micrograph software (both from Gatan Inc.). Micrographs were taken of peritubular capillaries, and fenestrations were quantified as explained below.

### Fluorescence microangiography.

Fluorescence microangiography was used to visualize perfused vessels as described previously (94). In brief, 500 μL FluoSpheres sulfate (0.02 μm; F8845; Thermo Fisher Scientific) was mixed with 4.5 mL low melting point agarose and kept at 41°C. All solutions were preheated to 41°C. Heart perfusion was carried out in dormicum/hypoderm-sedated mice starting with 1 mL heparinized saline (100 IU/mL; H3149; Sigma-Aldrich), followed by 1 mL 3 M KCl, 5 mL PBS, and 5 mL FluoSpheres/agarose mix. After perfusion, kidneys were dissected and put on ice for 20 min followed by fixation in 4% paraformaldehyde for 2 h. Tissue was stored in PBS at 4°C until further processing. For quantification, 100 μm vibratome sections were imaged in a Leica SP8 confocal microscope. The area of FluoSpheres perfusion was correlated to total vasculature (*Cdh5*-TdTomato) in each image.

### Image quantification.

For immunofluorescence analysis, kidney cortex was imaged (5 images/mouse, CL and UUO) at original magnification ×400 using a Leica SP8 confocal microscope. Analysis was focused on the cortex, as the medulla disappears at later time points after UUO. For estimation of fibrosis, aSMA^+^ and vimentin^+^ staining was quantified in each image using Otzu thresholding in ImageJ. Large arteries and glomeruli were excluded. Fibrotic area was expressed as a percentage of the whole image area. Quantification of vascular area was done in the same way as above for endomucin and podocalyxin and correlated to the total number of nuclei per image. The number of nuclei was counted using Analyze Particles in ImageJ with selection of positive areas of 50–800 pixels.

Proliferating endothelial cells (Ki67^+^ or EdU^+^) were counted in *Cdh5*-TdTomato^+^ nuclei in 5 image fields/mouse and correlated to the total number of endothelial nuclei per image (*Cdh5*-TdTomato^+^).

From electron micrographs, capillary fenestrations of peritubular and glomerular capillaries were semiquantitatively graded and expressed as a score between 0 and 4, with 0, 0%–5%; 1, 6%–25%; 2, 26%–50%; 3, 51%–75%; and 4, 76%–100% of the capillary length having fenestrations (i.e., a score of 4 in healthy kidneys), as previously described (16). To evaluate podocyte injury, the number of foot processes per glomerular basement membrane length was measured in ImageJ, and the foot process width was calculated as described previously (95).

An increased number of vacuoles was discovered during electron microscopy analysis and was therefore quantified on semithin sections stained with toluidine blue as the number of affected tubular segments correlated to the total number of tubular segments per image taken at an original magnification of ×100. A tubular segment was defined as affected when vacuoles were present in the whole epithelial cell, from the luminal to the basolateral side (Figure 5A).

For densitometric analysis of TIE2 phosphorylation, Image Lab software (Bio-Rad) was used, and results expressed correlated to total TIE2 (pTIE2/total TIE2).

### Human data.

We utilized the Nephroseq platform (Nephroseq.org) to obtain human data for *CDH5* (endothelial marker), *ANGPT2*, *PDGFB*, and *ANGPT1* from 2 different microarray datasets: renal biopsies with histopathological confirmed CKD (96) and renal biopsies from transplant patients with/without renal dysfunction (97). Data from living or cadaveric donors were combined. Data are presented as Log2 expression, and statistical differences were from Nephroseq.

### Bulk RNA-Seq and data analysis.

RNA was prepared from kidney tissue as above from all groups; IgG (*n* = 8), ABTAA (*n* = 7), *Veptp*^WT^ (*n* = 7), *Veptp*^KO^ (*n* = 8), *Pdgfb*^WT^ (*n* = 4), and *Pdgfb*^KO^ (*n* = 4). Two *Veptp*^KO^ CL and two *Pdgfb*^WT^ CL samples were lost in plate preparation. Kidneys from 2 sham-operated *Pdgfb*^WT^ mice were used in the *Pdgfb*^WT^ CL group. Samples were run in quadruplets in a 384-well format. Library preparation and sequencing were performed as described previously (98). The uniquely indexed cDNA libraries from the 384-well plate were pooled and sequenced together on the lane of a HiSeq 3000 sequencer (Illumina). The 4 raw fastq sequences for each quadruple sample were merged and mapped with a standard STAR pipeline (version 2.7.3a). The duplicated reads were filtered out using UMI-tools (version 1.1.4). The gene expression count summary was calculated using the featureCounts function from the Subread package (version 1.4.6-p5) (99).

DEGs were calculated by group comparisons utilizing the R edgeR package (version 3.40.2) (100), which applied an exact test between the groups (101). The raw *P* values were corrected for multiple testing using the FDR method. FDR of 0.05 was set as threshold for significant differential expression. The raw count data were normalized using the cpm method. Gene expression heatmaps were visualized using row *z* score calculated from cpm utilizing Heatmapper (102). Metascape v3.5.20240901 (70) was used for GO enrichment of biological processes with DEGs from ABTAA- and IgG-treated groups. Statistical enrichment was calculated using the hypergeometric test with Benjamini-Hochberg correction to adjust for FDRs with a minimum overlap of 3 genes, a cutoff of *P* < 0.01, and a minimum enrichment of 1.5. For translatability, the analysis was done for the corresponding human gene annotation. The top 20 enrichment GO terms were visualized with heatmaps based on –log10(*P*).

### Statistics.

GraphPad Prism 10 (GraphPad Software Inc.) was used for graphical representation and statistical analysis of data. Data are expressed as geometric mean ± SD. Means between groups were compared using unpaired 2-tailed Student’s *t* test (2 groups) or 1-way ANOVA with Tukey’s multiple-comparison post hoc test (≥3 groups). Many groups of data had an uneven distribution; therefore, data were natural log-transformed before statistical analysis as previously suggested (103). A *P* value of less than 0.05 was considered significant. Please see *Bulk RNA-Seq and data analysis* for details regarding analysis of bulk RNA-Seq data.

### Study approval.

All procedures were approved in advance by the Uppsala Committee of Ethics of Animal Experiments (permit numbers C110/13, C100/15, 5.8.18-04862/2020, 5.8.18-04862/2023, and 5.8.18-20319/2024) and were conducted according to guidelines established by the Swedish Board of Agriculture. ARRIVE guidelines were used for reporting.

### Data availability.

RNA-Seq data can be accessed from the National Center for Biotechnology Information’s Gene Expression Omnibus database under accession number GSE306281. All data used to support the findings of this study, except the RNA-Seq data, are included in the paper, and the supplemental information is available from a searchable database at https://heomics.shinyapps.io/Jeansson_lab_kidney_UUO/ Values for all data points in graphs are reported in the Supporting Data Values file (Supplemental Data File 5).

## Author contributions

MJ conceived the study. RP, AMH, LH, SN, and MJ participated in data acquisition, analysis, and interpretation. SEQ and CB provided key reagents and intellectual input. RP and MJ wrote the manuscript; all authors contributed to critical reading and editing.

## Funding support

This work is the result of funding from the Swedish Research Council and NIH, in whole or in part, and is subject to the NIH Public Access Policy. Through acceptance of this federal funding, the NIH has been given a right to make the work publicly available in PubMed Central.

- Novo Nordisk Foundation Pioneer Innovator (grant NNF23OC0086927 to MJ).
- European Foundation for the Study of Diabetes (EFSD-BI-2023 to MJ).
- Swedish Kidney Foundation (F2021-0061, F2022-0046, and F2023-0064 to MJ).
- Knut and Alice Wallenberg Foundation (2024-0177 to MJ and 2015-0030 and 2020-0057 to CB).
- Swedish Research Council (2012-865 to MJ and 2015-00550 to CB).
- Åke Wiberg Foundation (738866289 to MJ).
- Magnus Bergwall Foundation (2013-24942 and 2014-00055 to MJ).
- IGP Young Investigator Award (to MJ).
- The use of Veptp-floxed mice was made possible through core services and support from the Northwestern University George M. O’Brien Kidney Research Core Center (NU GoKidney), a program funded by NIH/National Institute of Diabetes and Digestive and Kidney Diseases (P30 DK114857).

## Supplementary Material

Supplemental data

Supplemental data set 1

Supplemental data set 2

Supplemental data set 3

Supplemental data set 4

Supplemental data set 5

Unedited blot and gel images

Supporting data values

## Version 1 09/15/2025

In-Press Preview

## Version 2 11/03/2025

Electronic publication
