# Supplementary material for: TIE2 activation by antibody-clustered endogenous angiopoietin-2 prevents capillary loss and fibrosis in experimental kidney disease
Source: J Clin Invest. 2025 Sep 15;135(21):e190286. doi: 10.1172/JCI190286 (PMC12578391; doi:10.1172/JCI190286)
Supplement: Supplemental data [file jci-135-190286-s306.pdf]

Supplemental material for:

TIE2 activation by antibody-clustered endogenous angiopoietin-2 prevents capillary loss and fibrosis in experimental kidney disease

Authors: Riikka Pietilä[1], Amanda Marks[1], Liqun He[1], Sami Nanavazadeh[1], Susan E Quaggin[2], Christer Betsholtz[1,3], and Marie Jeansson[1,3] *

Affiliations:

[1]Department of Immunology, Genetics and Pathology, Uppsala University; Uppsala, Sweden.

[2]Feinberg Cardiovascular Research Institute and Division of Nephrology and Hypertension, Northwestern University; Chicago, IL, USA.

[3]Department of Medicine Huddinge, Karolinska Institutet; Huddinge, Sweden.

*Corresponding author. Marie Jeansson, Department of Medicine Huddinge, Blickagangen 16, 141 62 Huddinge, Sweden. Phone +45 791679182. Email: marie.jeansson@ki.se

Table of Contents

SUPPLEMENTAL FIGURES 2
SUPPLEMENTAL TABLES 9

SUPPLEMENTAL FIGURES

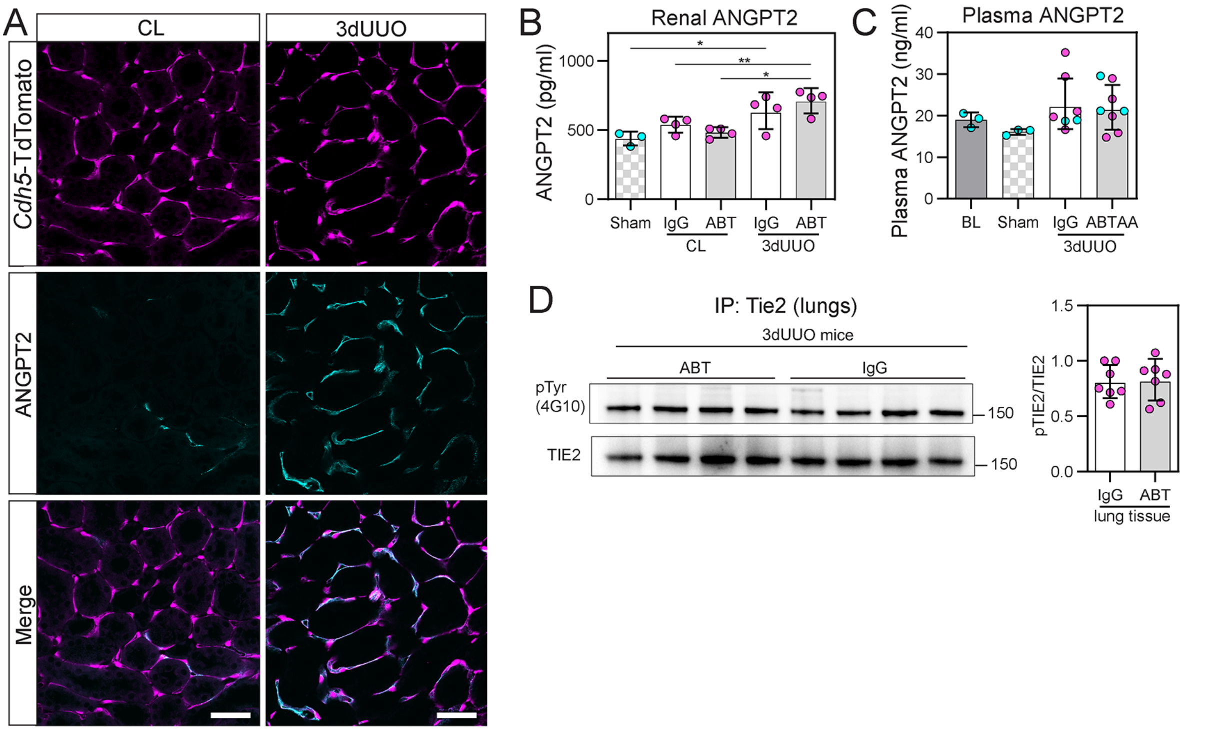

Supplemental Figure S1. ANGPT2 after UUO. (**A**) Confocal imaging of ANGPT2 staining (cyan) and *Cdh5*-TdTomato (magenta) in renal cortex of 3-day UUO kidneys. Representative images of n=3 mice. Scale bars, 50 µm. (**B**) ANGPT2 concentration in kidney homogenates in sham operated mice and 3dUUO and CL kidneys from IgG and ABTAA (ABT) treated mice. Data is based on n=3-4 mice/group. (**C**) Plasma concentrations of ANGPT2 at baseline (BL), in sham operated mice, and IgG or ABTAA treated 3-day UUO mice. Data based on n=3-8 mice/group. (**D**) pTIE2/TIE2 in lung tissue after ABTAA treatment 3-days after UUO. Data is based on n=7 mice/group and 2 blots, see Supplemental Figure S2. Data represent mean±SD and each symbol represents 1 mouse (females: magenta, males: cyan). *P<0.05, **P<0.01 with 1-way ANOVA and Tukey's post hoc test (**B**) or unpaired 2-tailed Student's t test (**D**).

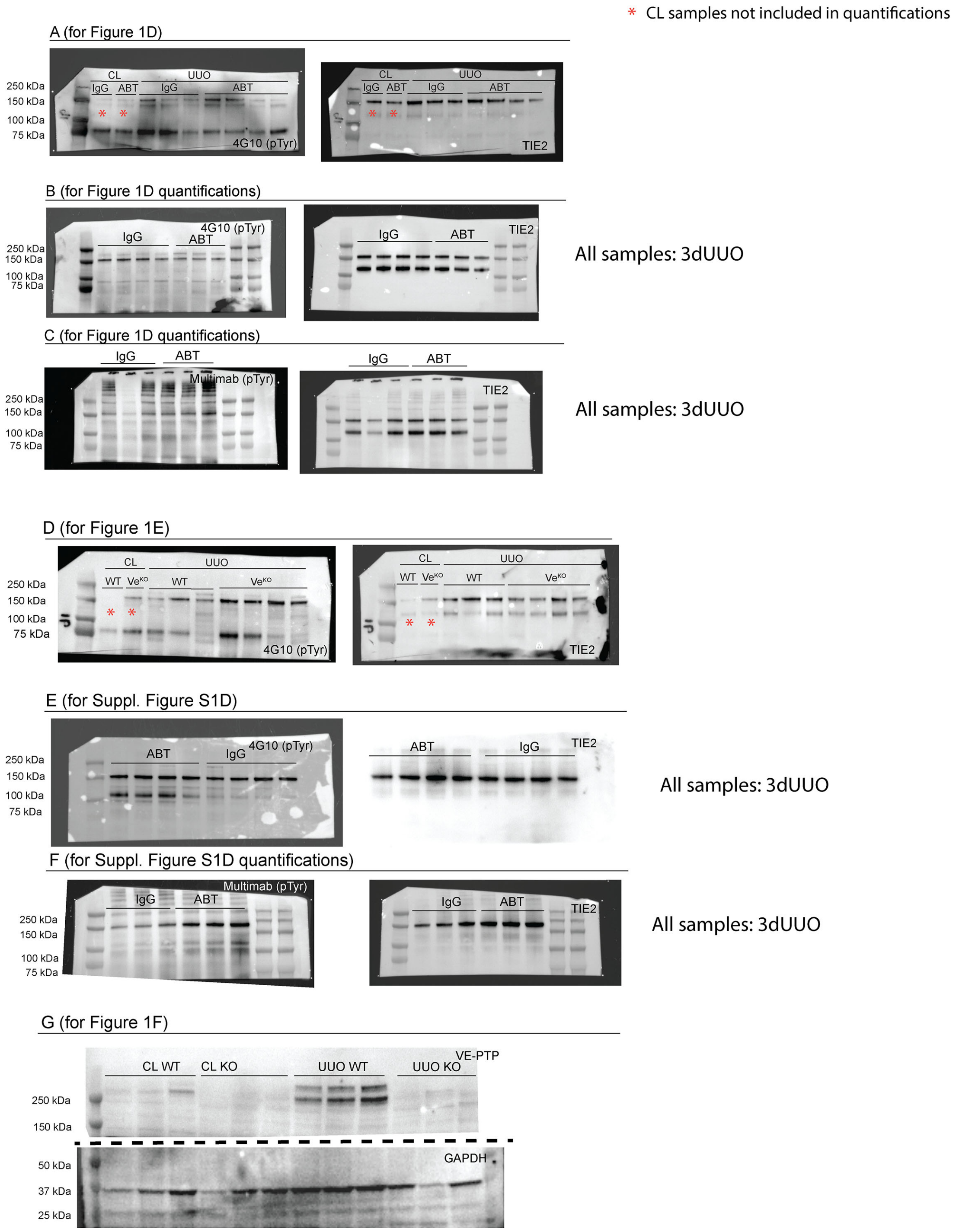

Supplemental Figure S2. Uncropped Western blot images. (**A-C**) Blot images from TIE2 IP of IgG and ABTAA treated kidneys 3 days after UUO. Blots show pTyr and TIE2 and were used for quantifications presented in Figure 1D. (**D**) Blot image from TIE2 IP of *Veptp*^{iECKO} and WT kidneys 3 days after UUO. Blot shows pTyr and TIE2 and was used for quantifications presented in Figure 1E. (**E** and **F**) Blot image from TIE2 IP of lung from IgG and ABTAA treated mice 3 days after UUO. Blots show pTyr and TIE2 and were used for quantifications in Figure 1D. (**G**) VEPTP blot image from *Veptp*^{iECKO} and WT kidneys used for quantification in Figure 1F. For visualization purposes, images of the immunostained membranes (**A-G**) are merged with corresponding calorimetric image of the molecular weight ladder Precision Plus Protein Dual Color Standards (1610394) seen on the left side, and prestained protein ladder (Ab1106128) on the right for (**F**).

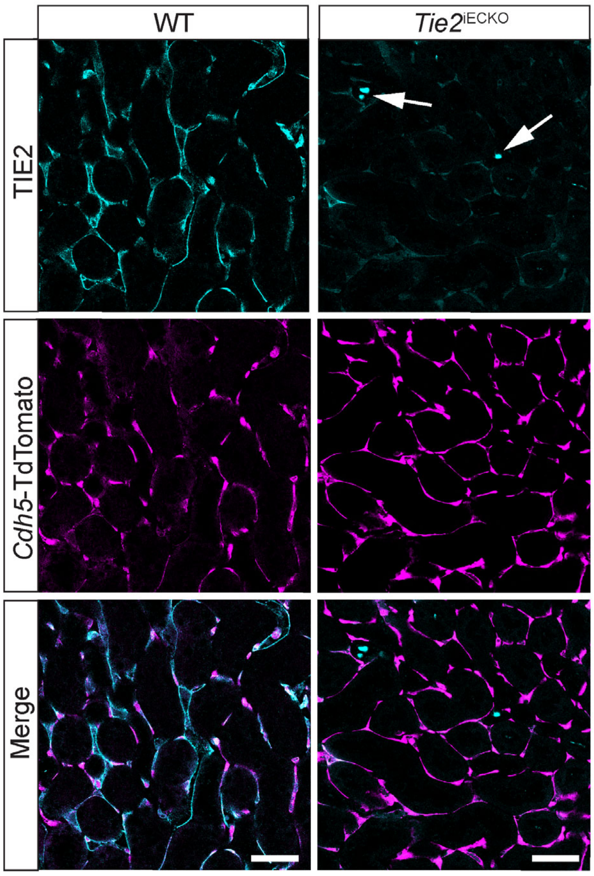

Supplemental Figure S3. Validation of *Tie2*^{iECKO} mice. Confocal imaging of renal cortex shows staining for TIE2 (cyan) and *Cdh5*-TdTomato reporter (magenta) in WT and *Tie2*^{iECKO} mice. Arrows indicate non-endothelial TIE2. Representative images of n=3/group. Scale bars, 50 µm.

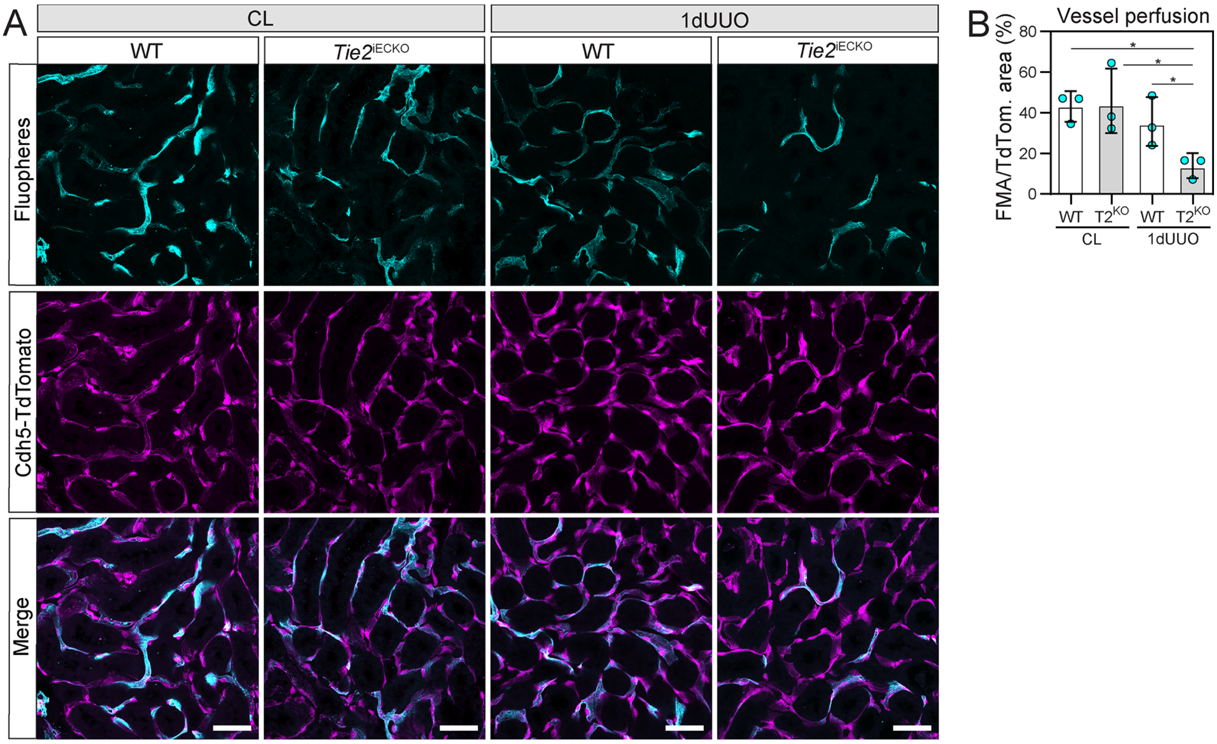

Supplemental Figure S4. Perfusion of peritubular capillaries after UUO. (**A, B**) Area quantifications and confocal images from renal cortex in 1-day UUO kidneys with fluorescence microangiopathy (FMA, cyan). Perfused vessels were correlated to the total vessel area (*Cdh5*-TdTomato, magenta). Data is based on n=3 mice/group and 60 images. Scale bars, 50 µm. Data represent mean±SD and, each symbol represents 1 mouse (females: magenta, males: cyan). *P<0.05 with 1-way ANOVA and Tukey's post hoc test.

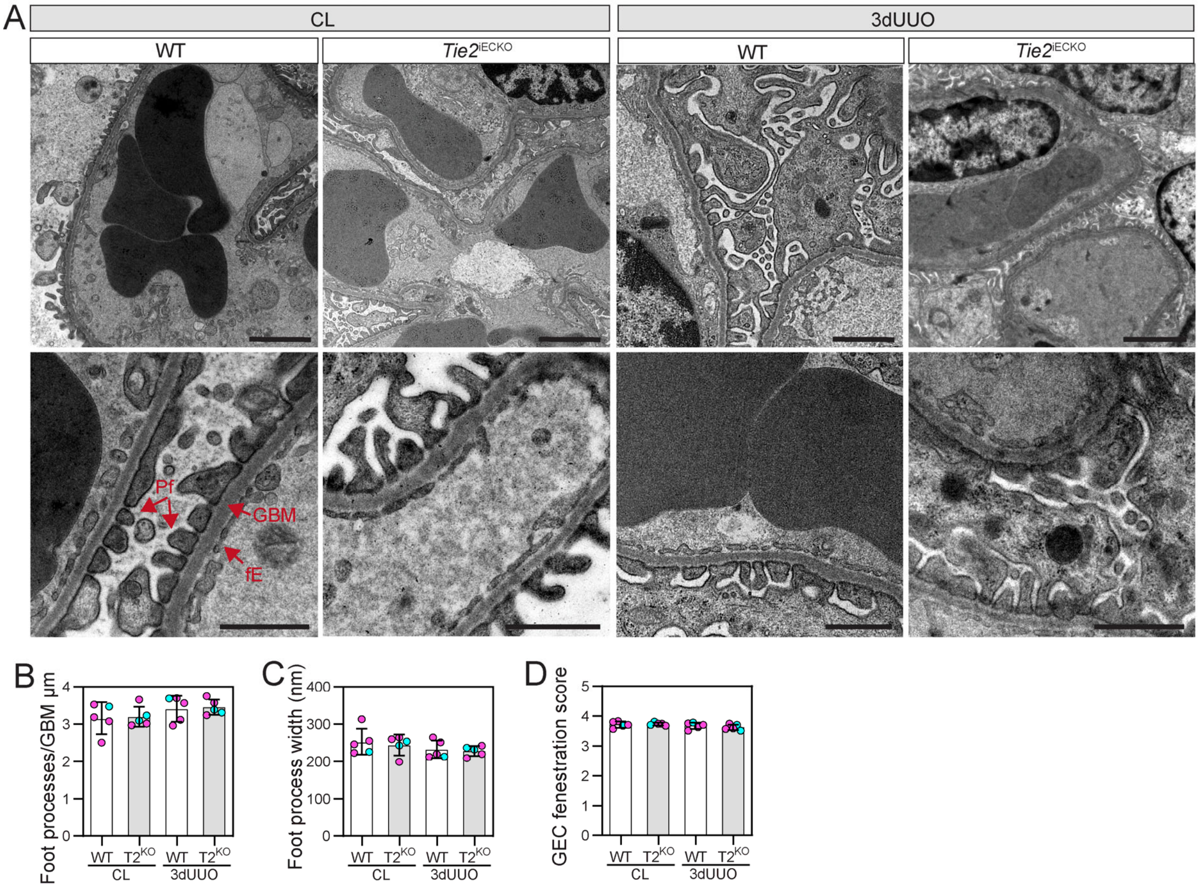

Supplemental Figure S5. Electron micrographs of glomeruli after UUO. (**A**) Electron micrographs of glomeruli 3 days after UUO in WT and *Tie2*^{iECKO} mice. Arrows indicate podocyte foot processes (Pf), fenestrated endothelium (fE), and glomerular basement membrane (GBM). Scale bar; upper panel, 10 µm, lower panel, 1 µm. (**B, C**) Evaluation of podocyte injury by measurement of the number of foot processes per GBM µm length and calculation of foot process width 3 days after UUO. Data is based on n=5 mice/group and >300 micrographs. (**D**) Evaluation of glomerular endothelial cell (GEC) fenestration score 3 days after UUO. Scoring based on percentage of endothelium with fenestrations; 0: 0-5%, 1: 6-25%, 2: 26-50%, 3: 51-75%, and 4: 76-100%. Data based on n=5 mice/group and >250 micrographs. Data represent mean±SD and each symbol represents 1 mouse (females: magenta, males: cyan). Statistical analysis with 1-way ANOVA and Tukey's post hoc test (ns).

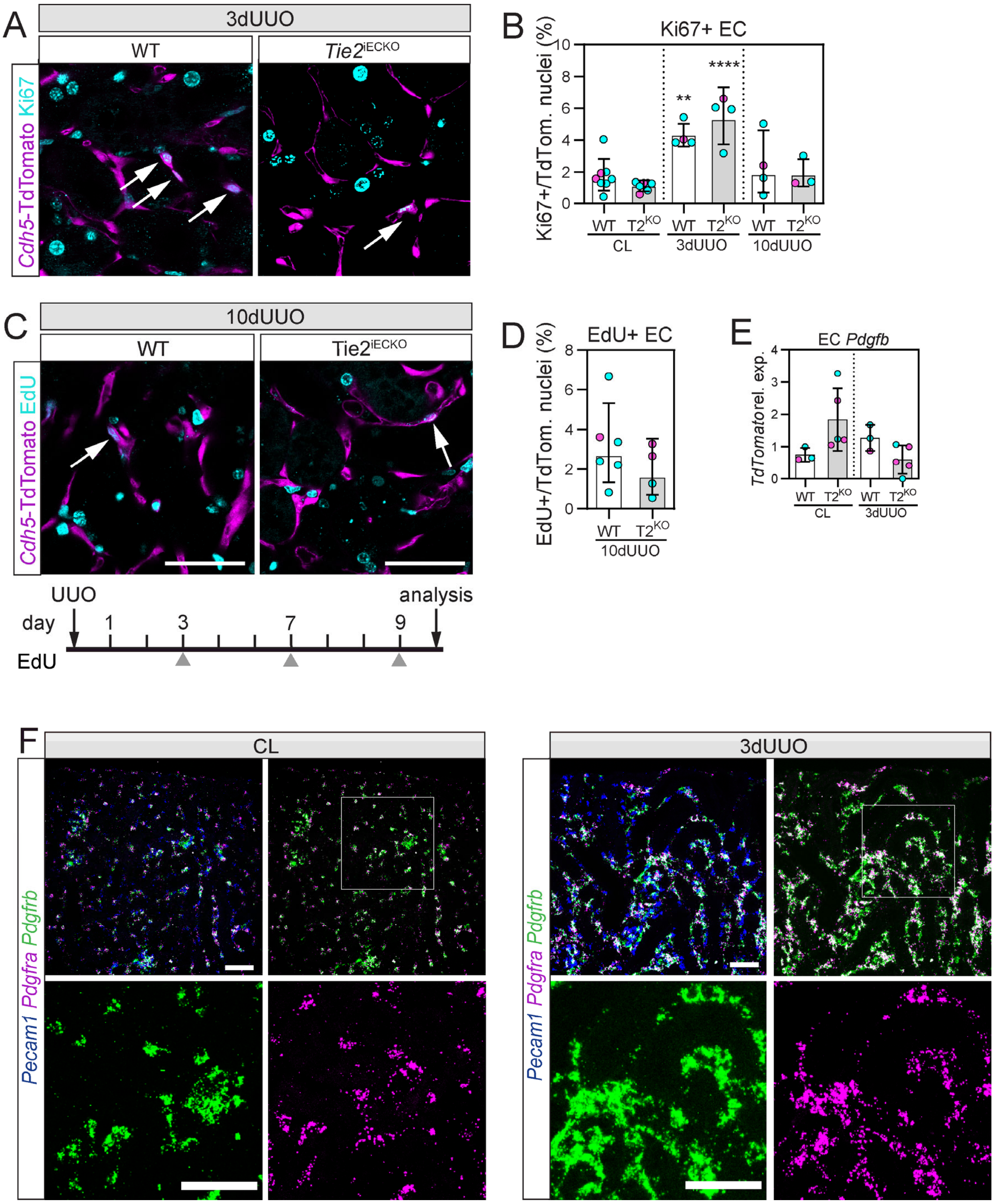

Supplemental Figure S6. Endothelial proliferation after UUO. (**A** and **B**) Staining and quantification of proliferating cells with Ki67 (cyan) together with *Cdh5*-TdTomato reporter (magenta) in renal cortex of 3-day UUO kidneys from WT and *Tie2*^{iECKO} mice. Data is based on n=3-4 mice/group and 150 images. (**C** and **D**) Staining and quantification of proliferating cells with EdU injected at indicated timepoints in renal cortex of 10-day UUO kidneys from WT and *Tie2*^{iECKO} mice. Data is based on n=4-6 mice/group and 50 images. (**E**) Expression for *Pdgfb* in isolated endothelial cells (*Cdh5*-TdTomato+) in 3-day UUO kidneys from WT and *Tie2*^{iECKO} mice. Cells were sorted directly into lysis buffer. Data is based on n=3-5 mice/group. (**F**) RNA-ISH for *Pecam1* (blue), *Pdgfra* (magenta), and *Pdgfrb* (green) in 3-day UUO kidneys. Representative image of n=3 mice. Scale bars, 50 µm. Data in graphs represent mean±SD and each symbol represents 1 mouse (females: magenta, males: cyan). $^{**}P<0.01$, $^{****}P<0.0001$ with 1-way ANOVA (**B** and **E**) and Tukey's post hoc test (**D**).

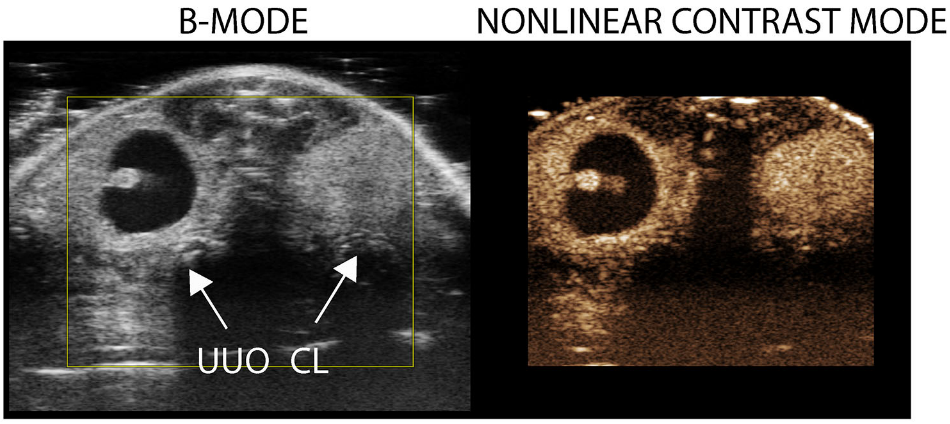

Supplemental Figure S7. Contrast imaging ultrasound. Images from ultrasound of CL and UUO kidneys in B-mode and nonlinear contrast mode.

SUPPLEMENTAL TABLES

Table S1: Genotyping primers

**Gene**	**Forward 5'-**	**Reverse 5'-**	**Size (bp)**
Tie2 wt	TCCTTGCCGCCAACTTGTAAAC	AGCAAGCTGACTCCACAGAGAAC	175
Tie2 flox	CACTTTCTACTCTGTTGAC	CCTTACATGTTTTACTAG	338
Cre	ATGTCCAATTTACTGACCG	CGCCGCATAACCAAGTGAA	673
TdTomato	CTGTTCCTGTACGGCATGG	GGCATTAAAGCAGCGTATCC	196
Veptp flox	GCGTCTATCCAGTGGAGGACTTTC	CCAGGTGCCGTTCATTCAGC	wt 489<br>lox 546
Pdgfb flox	TTTGAAGCGTGCAGAATGCC	GGAACGGATTTTGGAGGTAGTGTC	wt 265<br>lox 350

Table S2: Gender distribution for animal experiments.

Experiment	Total	Female Control	Female KO or ABTAA	Male Control	Male KO or ABTAA
**ABTAA, body weight***		20.9 ± 2.2	20.5 ± 2.0	25.2 ± 4.6	27.9 ± 4.1
3dUUO, *N*	44	13	10	5	16
10dUUO, *N*	11	3	3	3	2
Veptp$^{\mathbf{iECKO}}$**, body weight***		17.9 ± 1.7	18.6 ± 1.2	24.1 ± 3.4	25.9 ± 1.9
3dUUO, *N*	36	13	8	9	6
Tie2$^{\mathbf{iECKO}}$**, body weight***		20.3 ± 2.8	20.2 ± 2.9	27.41 ± 4.2	25.9 ± 4.0
1dUUO, *N*	35	6	5	13	11
3dUUO, *N*	72	21	19	15	17
10dUUO, *N*	25	7	9	7	2
Pdgfb$^{\mathbf{iKO}}$**; body weight***		22.7 ± 0.4	19.5 ± 4.2	28.7	27.6 ± 1.8
3dUUO, *N*	12	6	2	1	3
**Timeline, body weight***		18.7 ± 1.2		25.3 ± 1.1	
6-24 hours, *N*	21	9		12	
**Total**	256	78	56	65	57

* Mean ± SD body weight (g)

Table S3: Antibodies

Target	Catno	Lot	Company	Conc.
aSMA/ACTA2-Cy3	C6198	0000122898	Sigma	IHC 1:200
aSMA/ACTA2-Alexa 647	sc32251	J1518	Santa Cruz	IHC 1:200
Endomucin	Ab106100	GR3212325-1	Abcam	IHC 1:200
Vimentin	Ab92547	GR3186827-22	Abcam	IHC 1:200, WB 1:1000
Podocalyxin	AF1556	JPC0117081	R&D Systems	IHC 1:200
ANGPT2 (ABA)	from G.Y.K.*			IHC 1:200
VEPTP	from D.V#			WB 1:1000
TIE2	AF762	EFK021902A	R&D Systems	IHC 1:200, WB 1:1000
TIE2	19157-1-AP	00134014	Thermo Fisher Scientific	IP
Phospho-tyrosine G410	05-321	2202535	Merck	WB 1:1000
Ki67	Ab15580	GR3196371-1	Abcam	IHC 1:200
Donkey anti rabbit IgG – Alexa 680	A10043	2165747	Thermo Fisher Scientific	IHC 1:200
Donkey anti rabbit IgG-Alexa 555	A31572	1837922	Thermo Fisher Scientific	IHC 1:200
Donkey anti goat IgG-Alexa 633	A21082	2309146	Thermo Fisher Scientific	IHC 1:200
Donkey anti rat IgG-Alexa 488	A21208	1100239	Thermo Fisher Scientific	IHC 1:200
Donkey anti rat IgG- Alexa 633	A21094	2002975	Thermo Fisher Scientific	IHC 1:200
GAPDH-HRP	Ab9482		Abcam	WB 1:5000
ACTB-HRP	MA5-15739	XC341986	Thermo Fisher Scientific	WB 1:20 000
Rabbit IgG-HRP	sc-2357	I2022	Santa Cruz	WB 1:10 000
Rabbit IgG-HRP	A16096	94-22-070122	Invitrogen	WB 1:5000
Goat IgG-HRP	A16005	80-180-030322	Invitrogen	WB 1:10 000
Goat IgG-HRP	AP180P	3816313	Millipore	WB 1:5000
Mouse IgG-HRP	W402B	0000569731	Promega	WB 1:2500

Kind gift from *Gou Young Koh (G.Y.K) and #Dietmar Vestweber (D.V)

Table S4: Probes for real time PCR

**Gene**	**Probe**	**Company**
Hprt	Mm03024075_m1	Thermo Fisher Scientific
Gapdh	Mm99999915_g1	Thermo Fisher Scientific
Col1a1	Mm00801666_g1	Thermo Fisher Scientific
Tagln	Mm00441661_g1	Thermo Fisher Scientific
Fn1	Mm01256744_m1	Thermo Fisher Scientific
Angpt1	Mm00456503_m1	Thermo Fisher Scientific
Pdgfb	Mm00440677_m1	Thermo Fisher Scientific
Pdgfrb	Mm00435546_m1	Thermo Fisher Scientific
Tek/Tie2	Mm01256897_m1	Thermo Fisher Scientific
Havcr1	Mm00506686_m1	Thermo Fisher Scientific
Adgre1/F480	Mm00802529_m1	Thermo Fisher Scientific
