# Supplementary material for: TIE2 activation by antibody-clustered endogenous angiopoietin-2 prevents capillary loss and fibrosis in experimental kidney disease
Source: J Clin Invest. 2025 Sep 15;135(21):e190286. doi: 10.1172/JCI190286 (PMC12578391; doi:10.1172/JCI190286)
Supplement: Unedited blot and gel images [file jci-135-190286-s312.pdf]

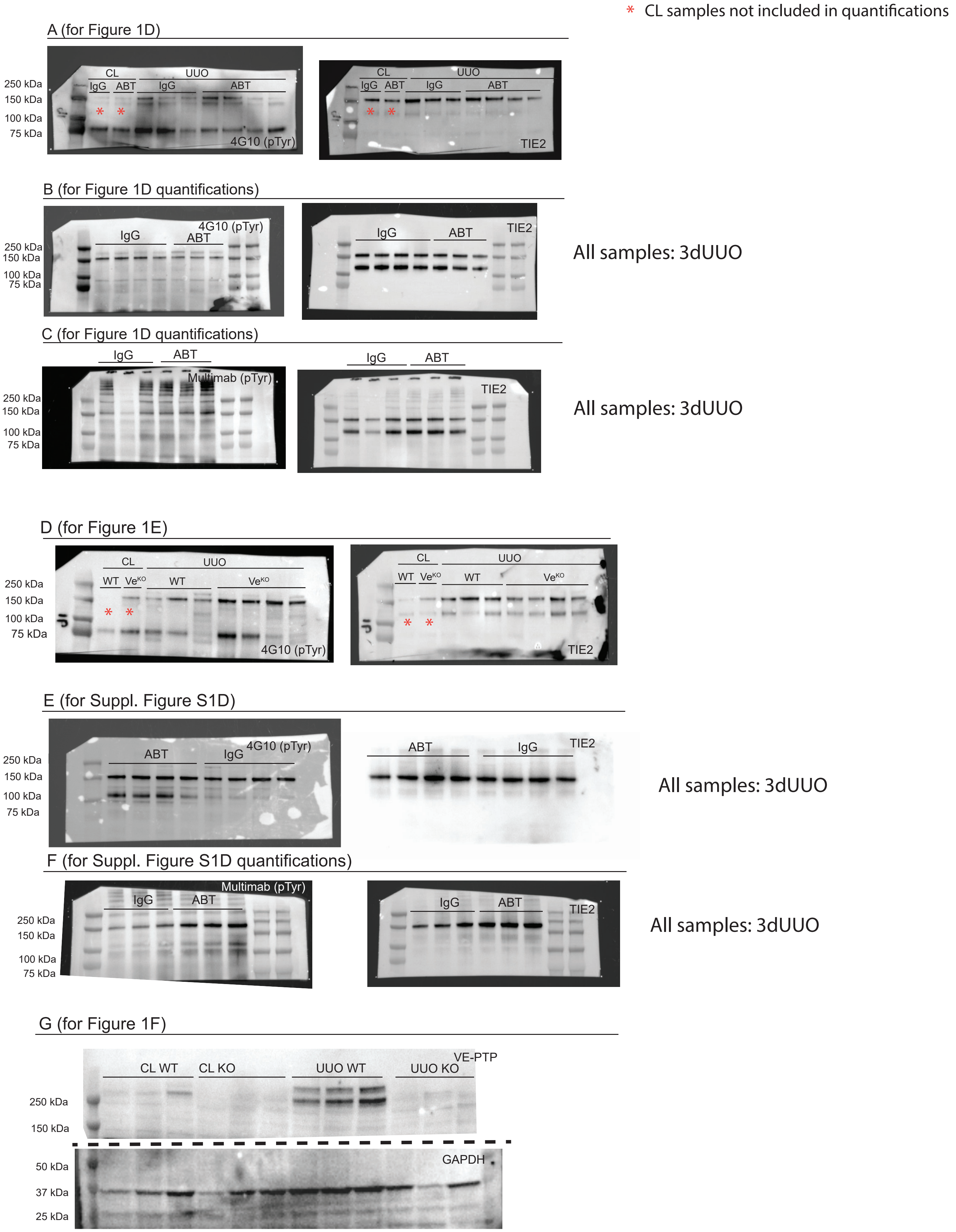

Uncropped western blot images. (A) Tie2 IP from IgG and ABTAA treated mice kidneys.
(B,C) Tie2 IP from IgG and ABTAA treated mice kidneys (two independent experiments). (D) Tie2 IP from VE-PTP WT and KO mice kidneys. (E) Tie2 IP from IgG and ABTAA treated mice lungs. (F) Tie2 IP from IgG and ABTAA treated mice lungs (independent experiment). (G) Western blot from VE-PTP WT and KO mice whole kidney lysates.
For visualization purposes, images of immunostained membranes are merged with colorimetric image of same membrane where molecular weight ladder (Precision Plus Protein™ Dual Color Standards, cat no #1610394) can be seen.
